# Acceptability and content validity of suicidality screening items: a qualitative study with perinatal women

**DOI:** 10.3389/fpsyt.2024.1359076

**Published:** 2024-04-11

**Authors:** Elizabeth Dudeney, Rose Coates, Susan Ayers, Rose McCabe

**Affiliations:** ^1^ Centre for Maternal and Child Health Research, School of Heath and Psychological Sciences, City, University of London, London, United Kingdom; ^2^ Centre for Mental Health Research, School of Heath and Psychological Sciences, City, University of London, London, United Kingdom

**Keywords:** suicide, perinatal, pregnancy, postpartum, screening, acceptability, qualitative, content analysis

## Abstract

**Background:**

Suicide is a leading cause of death for perinatal women. It is estimated that up to 50% of women with mental health issues during pregnancy and/or after birth are not identified, despite regular contact with healthcare services. Screening items are one way in which perinatal women needing support could be identified. However, research examining the content validity and acceptability of suicide-related screening items with perinatal women is limited.

**Aims:**

This study sought to: (i) assess the acceptability and content validity of 16 suicide-related items that have been administered and/or validated in perinatal populations; and (ii) explore the potential barriers and facilitators that may affect how women respond to these items when administered during pregnancy and after birth.

**Methods:**

Twenty-one cognitive and semi-structured interviews were conducted with pregnant and postnatal women in the UK. The sample included women who had experienced self-reported mental health problems and/or suicidality during the perinatal period, and those who had not. Interviews were transcribed verbatim, and a coding framework based on the Theoretical Framework of Acceptability was applied to explore the data using deductive and inductive approaches.

**Results:**

Findings indicated that the acceptability and content validity of suicide-related items were largely unacceptable to perinatal women in their current form. Women found terms such as ‘better off dead’ or ‘killing myself’ uncomfortable. Most women preferred the phrase ‘ending your life’ as this felt less confronting. Comprehensibility was also problematic. Many women did not interpret ‘harming myself’ to include suicidality, nor did they feel that abstract language such as ‘leave this world’ was direct enough in relation to suicide. Stigma, fear, and shame was central to non-disclosure. Response options and recall periods further affected the content validity of items, which created additional barriers for identifying those needing support.

**Conclusions:**

Existing suicide-related screening items may not be acceptable to perinatal women. Maternity practitioners and researchers should consider the phrasing, clarity, context, and framing of screening items when discussing suicidality with perinatal women to ensure potential barriers are not being reinforced. The development of specific suicidality screening measures that are acceptable, appropriate, and relevant to perinatal women are warranted.

## 1 Introduction

Maternal suicide is a devastating global issue, accounting for up to one fifth of deaths during the perinatal period in high-income countries (HICs) (1–4) and contributing to pregnancy-related mortality in lower- and middle-income countries (LMICs) (5). In the UK, suicide is the leading direct cause of death for women between six-weeks and one-year post pregnancy, a figure that has tragically remained largely unchanged for over a decade (6, 7). Furthermore, the incidence of maternal death due to mental health related causes has increased over the past few years, now accounting for almost 40% of all deaths during the perinatal period, with many women having experienced multiple adversities (7, 8). Evidence also indicates that approximately half of women suffering from mental health problems and/or those at risk of suicide during the perinatal period are not identified, despite regular contact with maternity services (9, 10). It is therefore imperative that multi-sector approaches for understanding and addressing the potential risk factors and barriers that might prevent perinatal women from disclosing suicidal thoughts and/or behaviours are established. Such efforts may help to lessen the occurrence of preventable maternal deaths and improve outcomes for both women and their babies.

Pregnant women and new mothers are now recognised as a high-risk priority group in the UK cross-government suicide prevention plan for England (11). This strategy aims to reduce suicide rates through targeted and tailored interventions over the next five-years. The most recent report highlighted the importance of screening for mental health problems during pregnancy and after birth and suggested that care providers take an active role in exploring the risk factors for suicide at every perinatal contact. However, universal screening for common perinatal mental health problems is not currently recommended in the UK (12), despite evidence that this approach can lead to both a reduction in perinatal depression and anxiety symptoms (13) and is associated with increased referral rates and engagement with the appropriate services (14). Instead, the National Institute for Health and Care Excellence (NICE) (15) suggests that midwives ask about women’s mental health and wellbeing at their first antenatal booking appointment and at all subsequent contacts throughout the perinatal period using the Whooley questions (16) and/or the Generalized Anxiety Disorder Scale-2 (GAD-2) (17, 18). If a woman positively endorses either of these measures, a further assessment using psychometric self-report measures such as the Edinburgh Postnatal Depression Scale (EPDS) (19) or Patient Health Questionnaire-9 (PHQ-9) (20) can be conducted.

However, suicidal ideation and/or self-harm ideation is not routinely discussed with women receiving maternity care unless there is pretext for it, e.g., a prior history of suicidal and/or harming behaviours, if the woman is already receiving specialist care, or has self-disclosed. To date, no self-report screening measures of suicidality have been specifically designed for use with pregnant and postnatal women. When there is concern for a woman’s mental health, the presence or absence of suicidal and/or self-harm ideation is often identified in the context of screening for depression and/or other common mental health problems because: (i) suicidality and depression are frequently comorbid (2, 21); (ii) depression is a risk factor for suicide (22); (iii) many depression measures include an item that asks about suicidal and/or self-harming ideation (23); and (iv) screening measures are relatively quick to administer and complete. However, although depression screening has become a proxy for identifying possible suicidality in perinatal women, it is important that the broader implications of this approach are not overlooked. Whilst depression and suicidal ideation and/or suicide behaviours do overlap, suicidality can occur without the presence of depression (and vice versa), thus screening for perinatal suicidality using depression measures alone may result in cases being missed (24–26). Furthermore, suicide is a multifaceted phenomenon, characterised by a highly complex interplay of biology, psychology, environment, and culture (27). Hence, using a single item to capture the presence or absence of suicidal ideation and/or suicidal behaviours has clear limitations. Approaches for identifying suicidality need to consider numerous psychosocial factors as part of a comprehensive assessment, and distinguish between suicidal ideation (which can include both passive thoughts about a desire to die, and/or active thoughts about ending your own life by suicide), suicide plans, and suicide behaviours because these are distinct processes, with differing implications in terms of mitigating risk and developing individual care plans (28). NICE (29) do not recommend that risk assessment tools are used to predict future suicide, nor should they be used to determine who is offered treatment or not.

Psychometric validity and reliability of tools for identifying mental health problems in pregnancy and after birth needs to be rigorously tested in diverse settings and contexts (30). However, a recent systematic review (23) indicated that the validity and reliability of measures that have been used to identify suicidality in perinatal women is limited, and nearly all were either items or subscales on a measure for depression. Research should continue to evaluate the psychometric properties of suicidality measures in perinatal populations to ascertain the suitability of their use. It is equally important to explore the content validity and acceptability of screening measures to perinatal women because this may affect their appropriateness and uptake. Content validity is generally assessed by looking at how well a measure captures the construct(s) it is meant to represent, particularly in terms of its relevance, comprehensiveness, and comprehensibility to the target population (31). However, approaches for defining and assessing acceptability are more varied. Whilst it is now widely recognised that acceptability should be a key consideration in the development, evaluation, and implementation of healthcare interventions, there has been a lack of agreement or guidance regarding how it should be measured, and the use of existing theory is often overlooked (32). These inconsistencies can inhibit the comparison of acceptability across intervention types and settings, and limit evidence-based policy and or practice recommendations.

Research has examined the acceptability of perinatal mental health interventions and/or screening using various methods, including: (i) semi-structured interviews/focus groups to explore experiences, attitudes and/or perceptions (33); (ii) cognitive interviews to measure comfort, ease, recall, and confidence in answering depression case-finding questions (34); (iii) self-report surveys/questions to measure usefulness, comfort etc (35); and, (iv) uptake as an indicator of acceptability (36). Perinatal mental health assessment and commonly used measures appear to be acceptable to pregnant and postnatal women given certain conditions (37–39). For example, care providers need to explain the purpose, outcome, and follow-up procedures of mental health screening to perinatal women because this can influence their willingness to engage (40). Likewise, factors such as the mode of administration, individual comfort levels, relationship with healthcare professionals, and stigma, guilt, shame, and fear associated with perinatal mental health problems can affect acceptability and prevent women from answering screening measures honestly, which creates additional barriers for identifying those who might require support (39, 41–46).

Considering these barriers, it is unsurprising that identifying suicidality in perinatal women poses an even greater challenge. This is further exacerbated by the complex risk factors associated with suicidal ideation and behaviours (47, 48) and mitigating women’s fears regarding the potential consequences of disclosing suicidality e.g., unwanted intervention, hospitalisation, and/or concerns that their child will be taken away. Whilst there are a few studies that have explored and theorised women’s experiences of suicidality during the perinatal period (49–52), there is little literature that has specifically examined how pregnant and postnatal women feel about suicide-related items that are embedded into mental health screening measures or how acceptable these items are to them. Some studies have found that perinatal women from LMICs are hesitant to answer item-9 from the PHQ-9 (“Thoughts that you would be better off dead or of hurting yourself in some way”) due to religious beliefs (53) and/or because they find it uncomfortable (54), and other research has highlighted that there may be ambiguity regarding item-10 from the EPDS (“The thought of harming myself has occurred to me”) because this may not be interpreted to include suicidal ideation (37, 55). Furthermore, there is no universal consensus regarding the operational definition of suicidal ideation, which may create confusion in terms of identifying passive or active intent (56), and variations in language use/the phrasing of items may also play a significant role in perpetuating or reducing stigma and fear, and/or addressing cultural differences.

Therefore, given that the acceptability of suicide-related screening items is understudied in perinatal populations, and that research using an existing theory of acceptability to explore perinatal mental health screening measures is limited, it is important to adopt an established and structured approach to conducting research in this area to ensure methodological rigour. This will help to generate new knowledge and contribute to the systematic enquiry of acceptability in the perinatal mental health and suicide literature. The Theoretical Framework of Acceptability (TFA) (32) offers a systematic and evidence-based approach for evaluating the acceptability of healthcare interventions, from both the perspective of those receiving and/or those delivering the intervention. The TFA defines acceptability as a multifaceted construct, comprising of seven domains (affective attitude, burden, ethicality, intervention coherence, opportunity costs, perceived effectiveness, and self-efficacy, see **Table 1** for construct definitions) that can be applied at different timepoints (before, during or after an intervention) to assess the prospective, concurrent, or retrospective acceptability of an intervention. Perinatal research has used the TFA to assess acceptability in a variety of healthcare contexts (e.g., 57–61), including the use of exposure therapy among pregnant women with elevated anxiety (62), and a systematic review on the acceptability of implementing patient reported measures in routine maternity care (63). However, the authors know of no research that has used the TFA to assess the acceptability of suicide-related screening items to perinatal women. Using the TFA should provide valuable insights regarding the content validity of these items for identifying suicidal ideation and/or suicidal behaviours in perinatal women and it might also highlight some of the barriers and facilitators that influence how women respond to them.

**Table 1:** Constructs of the adapted Theoretical Framework of Acceptability (TFA) (32).

TFA construct	Definition Sekhon et al. (32)	Definition as applied to this study
Affective attitude	How an individual feels about the intervention	How an individual feels about the item (e.g., do they feel comfortable with the item, do they like the phrasing of the item, is the item distressing in any way)
Burden^1^ Intervention coherence^1^	The perceived amount of effort that is required to participate in the intervention The extent to which the participant understands the intervention and how it works	The amount of (cognitive) effort required to understand the item and how it works (e.g., is the item clear or confusing in any way, do they find the item too difficult to answer, is it a good fit for asking about suicidality)
Opportunity costs^2^ Self-efficacy^2^ Ethicality^2^	The extent to which benefits, profits or values must be given up by engaging in the intervention The participant’s confidence that they can perform the behaviour(s) required to participate in the intervention The extent to which the intervention has a good fit with an individual’s value system	How confident a participant is that they could answer this item (and/or answer it honestly) in light of their own value systems, motivation, and the potential costs/benefits of their response (e.g., is there anything that would prevent them from answering this item)
Perceived effectiveness	The extent to which the intervention is perceived as likely to achieve its purpose	The extent to which the item is perceived as likely to serve its purpose (e.g., do they think this item is useful or effective for identifying suicidality in perinatal women)

Superscript numbers ^(1,2)^ indicate that theses TFA constructs were clustered in this research.

Hence, the aims of this study were to: (i) assess the acceptability and content validity of 16 different suicide-related items that have been administered and/or validated in perinatal populations using the TFA; and (ii) explore the potential barriers and facilitators that may affect how women respond to these items when they are administered during pregnancy and after birth. These items were specifically selected from self-report measures for depression and/or common mental health problems because they have previously been used in perinatal populations as either a single item or as a subscale to identify suicidal ideation and/or behaviours (23). It is therefore important to assess how acceptable their use is to perinatal women, for both clinical and research purposes.

## 2 Materials and methods

### 2.1 Design and participants

This was a qualitative study using cognitive interviewing and semi-structured interviews to examine the acceptability and content validity of existing suicide-related screening items to perinatal women. All interviews took place online (e.g., Microsoft Teams or Zoom) or via the telephone, depending upon the participants preference.

The sample size for this study was guided by the Information Power approach (64). This approach suggests that five key areas should be considered when establishing a sample size estimation: (i) study aim(s); (ii) sample specificity; (iii) use of established theory; (iv) quality of dialogue; and (v) analysis strategy. It was anticipated that approximately 20 women would be suitable to meet the sample size requirements above. The final sample consisted of 21 participants.

Participants were pregnant and postnatal women (up to two-years postnatal), aged 18 or over, living in the United Kingdom, who were able to speak and understand English. The sample included both women who had experienced mental health problems and/or suicidality during the perinatal period in the past, and those who had not. Women who were experiencing current (self-reported) suicidality were not eligible to take part.

### 2.2 Recruitment and data collection

Of the 21 participants, most (*n* = 16) were recruited via social media (e.g., Twitter/X), and five were recruited via word of mouth. After making initial contact with the lead researcher (ED), eligible participants were sent an information pack which included a consent form, participant information sheet, and resource list to enable an informed decision about participation in the study. Once a participant had provided their informed consent, a convenient time was scheduled for them to take part in a one-to-one interview with ED, who was experienced in conducting qualitative interviews on sensitive topics and in identifying and signposting suicide risk.

A topic guide was developed by ED and RC, which comprised two parts. The first part of the interview used cognitive interviewing techniques to examine the acceptability and content validity of 16 suicide-related items taken from seven different depression and/or mental health screening measures, which have previously been administered and/or validated in perinatal populations (23). Participants were asked to sequentially read out each item (and its corresponding response options) and to ‘think aloud’ when verbalising their thoughts on the item to the researcher. Participants were not asked to answer the screening items directly, as our interest was in their thoughts about the application of these items in the ‘real world’ context, not in their responses to them per se. The researcher also used relevant probes to further explore the TFA constructs (as outlined in **Table 1**). The second part of the interview was a semi-structured interview about the broader implications of suicidality screening during the perinatal period. Detailed findings from this part of the interview are reported elsewhere.

Interviews lasted approximately 90 minutes, were audio-recorded, and transcribed verbatim and deidentified. Participants were reminded throughout the study that their participation was entirely voluntary and that they could withdraw from the study at any stage, up until the point of data analysis, without needing to provide a reason. All participants completed their interviews in full, with no obvious or reported adverse effects.

### 2.3 Measures

The 16 suicide-related items were taken from seven different screening measures of psychological distress, depression, and anxiety. Items were chosen because they have previously been administered and/or validated in perinatal populations to identify suicidal ideation and/or suicidal behaviours (23). A descriptive summary of the included measures/items is presented in **Table 2**. Six items were from the Inventory of Depression and Anxiety Symptoms (IDAS) (66) suicidality subscale. Five items were from the Postpartum Depression Screening Scale (PDSS) (67) suicidal thoughts scale. One item each was taken from: the Edinburgh Postnatal Depression Scale (EPDS) (19); the Patient Health Questionnaire-9 (PHQ-9) (20); the Self-Reporting Questionnaire-20 (SRQ-20) (68); the Beck Depression Inventory (BDI) (65); and the Ultra-Short Maternal Mental Health Screen (Ultra-Short) (69).

**Table 2:** Descriptive summary of measures.

Measure, item	Description of measure	Item content	Response options [scoring]	Recall period
Beck Depression Inventory (BDI), item-9*	21-item scale to screen for depression symptoms and severity	“I don’t have any thoughts of killing myself”, “I have thoughts of killing myself, but I would not carry them out”, “I would like to kill myself”, “I would kill myself if I had the chance”	Choose one of the statement options [0 to 3]	Past seven-days
Edinburgh Postnatal Depression Scale (EPDS), item-10*	10-item scale to screen for postnatal depression symptoms and severity	“The thought of harming myself has occurred to me”	“Never” [0], “Hardly ever” [1], “Sometimes” [2], “Yes, quite often” [3]	Past seven-days
Inventory of Depression and Anxiety Symptoms (IDAS), item-7*	64-item scale to screen for depression and anxiety symptoms	“I had thoughts of suicide”	“Not at all” [1], “a little bit” [2], “moderately” [3], “quite a bit” [4], “extremely” [5]	Past two-weeks
Inventory of Depression and Anxiety Symptoms (IDAS), item-9*	64-item scale to screen for depression and anxiety symptoms	“I hurt myself purposely”	“Not at all” [1], “a little bit” [2], “moderately” [3], “quite a bit” [4], “extremely” [5]	Past two-weeks
Inventory of Depression and Anxiety Symptoms (IDAS), item-14*	64-item scale to screen for depression and anxiety symptoms	“I thought about my own death”	“Not at all” [1], “a little bit” [2], “moderately” [3], “quite a bit” [4], “extremely” [5]	Past two-weeks
Inventory of Depression and Anxiety Symptoms (IDAS), item-15*	64-item scale to screen for depression and anxiety symptoms	“I thought about hurting myself”	“Not at all” [1], “a little bit” [2], “moderately” [3], “quite a bit” [4], “extremely” [5]	Past two-weeks
Inventory of Depression and Anxiety Symptoms (IDAS), item-41*	64-item scale to screen for depression and anxiety symptoms	“I cut or burned myself on purpose”	“Not at all” [1], “a little bit” [2], “moderately” [3], “quite a bit” [4], “extremely” [5]	Past two-weeks
Inventory of Depression and Anxiety Symptoms (IDAS), item-43*	64-item scale to screen for depression and anxiety symptoms	“I thought that the world would be better off without me”	“Not at all” [1], “a little bit” [2], “moderately” [3], “quite a bit” [4], “extremely” [5]	Past two-weeks
Patient Health Questionnaire-9 (PHQ-9), item-9*	9-item scale to screen for depression symptom prevalence and severity	“Have you had thoughts that you would be better off dead, or of hurting yourself in some way?”	“Not at all” [0], “several days” [1], “more than half the days” [2], “nearly every day” [3]	Past two-weeks
Postpartum Depression Screening Scale (PDSS), item-7*	35-item scale to screen for postnatal depression symptoms	“I started thinking that I would be better off dead”	“Strongly disagree” [1], “disagree” [2], “neither disagree nor agree” [3], “agree” [4], “strongly agree” [5]	Past two-weeks
Postpartum Depression Screening Scale (PDSS), item-14*	35-item scale to screen for postnatal depression symptoms	“I’ve thought that death seemed like the only way out of this living nightmare”	“Strongly disagree” [1], “disagree” [2], “neither disagree nor agree” [3], “agree” [4], “strongly agree” [5]	Past two-weeks
Postpartum Depression Screening Scale (PDSS), item-21*	35-item scale to screen for postnatal depression symptoms	“I wanted to hurt myself”	“Strongly disagree” [1], “disagree” [2], “neither disagree nor agree” [3], “agree” [4], “strongly agree” [5]	Past two-weeks
Postpartum Depression Screening Scale (PDSS), item-28*	35-item scale to screen for postnatal depression symptoms	“I felt that my baby would be better off without me”	“Strongly disagree” [1], “disagree” [2], “neither disagree nor agree” [3], “agree” [4], “strongly agree” [5]	Past two-weeks
Postpartum Depression Screening Scale (PDSS), item-35*	35-item scale to screen for postnatal depression symptoms	“I just wanted to leave this world”	“Strongly disagree” [1], “disagree” [2], “neither disagree nor agree” [3], “agree” [4], “strongly agree” [5]	Past two-weeks
Self-Reporting Questionnaire-20 (SRQ-20), item-17	20-item scale to screen for mental disorders	“Has the thought of ending your life been on your mind?”	YES/NO [1/0]	Past 30-days
Ultra-short Maternal Mental Health Screen (Ultra-Short), item-4	4-item scale to measure common perinatal mental disorders	“Has the thought of committing suicide often occurred to you?”	YES/NO [1/0]	Past month

* measure has been validated in perinatal populations.

Measures: Beck Depression Inventory (BDI) (65); Edinburgh Postnatal Depression Scale (EPDS) (19); Inventory of Depression and Anxiety Symptoms (IDAS) (66); Postpartum Depression Screening Scale (PDSS) (67); Patient Health Questionnaire-9 (PHQ-9) (20); Self-Reporting Questionnaire-20 (SRQ-20) (68); Ultra-Short Maternal Mental Health Screen (Ultra-Short) (69).

### 2.4 Data analysis

The deidentified transcripts were uploaded to NVivo 14 (70) for analysis. Using principles of the framework approach (71, 72), data were initially analysed using deductive content analysis, which is suitable for interpreting qualitative data in line with an existing framework or theory.

#### 2.4.1 Theoretical framework of acceptability

Following a familiarisation of the interview transcripts, ED and RC developed a coding framework based on the TFA constructs to inform the analysis. Sekhon et al. (32) suggest that the seven constructs may cluster or influence each other (Figure 3, p.8). Based on the initial coding, the decision was made to cluster the constructs of ‘burden and intervention coherence’ into one unique domain, and ‘opportunity costs, self-efficacy, and ethicality’ into another. Here, the construct of ‘burden’ was interpreted to be less associated with how much time or expense was required to answer each individual suicide-related item (as these were relatively short) and more related to the amount of cognitive burden (e.g., effort) that was required to understand the item and how it worked (‘intervention coherence’). The constructs of ‘opportunity costs, self-efficacy, and ethicality’ were clustered because together they related to how confident a participant might feel answering the item (and/or answering it honestly) in light of their individual value systems and the potential costs or benefits that may be associated with their response. ‘Affective attitude’ and ‘perceived effectiveness’ remained as single constructs. By conceptualising the constructs in this way, the researchers were able to assess the concurrent acceptability (e.g., as experienced in real time) and the prospective acceptability (e.g., if applied in the real world) of each suicide-related item in relation to the research aims. Definitions of the TFA constructs as applied to this study are presented in **Table 1**. In addition, the categories of ‘response options’ and ‘recall period’ were also included in the coding framework as relevant aspects of content validity.

The indexing procedure for each transcript involved: (i) selecting the data associated with the first suicide-related item (e.g., EPDS, item-10) and coding it under a corresponding heading (e.g., EPDS, item-10); (ii) coding all occurrences related to each of the framework categories for the item under the corresponding heading (e.g., ‘affective attitude’, ‘perceived effectiveness’ etc.); and (iii) applying positive (+), negative (–) and/or neutral/indifferent (+/-) codes to the data within each category. This process was repeated for all 16 suicide-related items across each transcript, and the data were then extracted into a matrix format using an Excel spreadsheet. This enabled the researchers to examine the number of positive, negative, and neutral/indifferent coding instances per item across all constructs and participants. Lastly, inductive coding was also applied to the qualitative data within each of the positive, negative, and neutral/indifferent categories to explore and generate themes within each of the domains.

The entire dataset was initially coded by ED and RC, with AZ independently coding 20% of the transcripts (adhering to the procedure above). Any areas of contention were discussed, and all minor coding discrepancies were revised as needed to ensure the trustworthiness of the analysis. ED and RC met regularly throughout the analytic process to discuss and review the coding as it progressed, and all coauthors agreed on the finalised main themes without disagreement. Reporting follows the guidelines set by the Consolidated Criteria for Reporting Qualitative research (73).

## 3 Results

### 3.1 Sample characteristics

Twenty-one perinatal women took part in this study. Three were pregnant at the time of their interview and 19 had a child under the age of two. One participant was both pregnant and had a child under the age of two. For 14 women, this was either their only child or first pregnancy. Most women were in their 30s (range 29 – 42 years, mean age 33.9). Twenty women spoke English as their first language and the cultural background of participants was predominantly White British. Nineteen women held a bachelor’s degree or higher qualification. Participant demographics are presented in **Table 3**.

**Table 3:** Sample characteristics (*n* = 21).

Sociodemographic variable	*M (range)* or *n* (%)
Age	33.9 (29 – 42 years)
Perinatal phase (at time of interview) Pregnancy Postnatal (≤ 24-months) Pregnant and postnatal*	2 (9%) 18 (86%) 1 (5%)
Number of additional children 0 1 2	14 (67%) 4 (19%) 3 (14%)
Mental health problems and/or suicidal thoughts during most recent pregnancy and/or after birth Yes No	17 (81%) 4 (19%)
Mental health problems and/or suicidal thoughts at any other time in life Yes No	13 (62%) 8 (38%)
Treatment and/or intervention for mental health problems at any time in life (including perinatal period) Yes No Not applicable	15 (72%) 3 (14%) 3 (14%)
English as first language Yes No	20 (95%) 1 (5%)
Education Secondary school (e.g., GCSE, SVQ level 1) Post-secondary (e.g., A-level, National Diploma Bachelor’s degree, or equivalent Master’s degree, or equivalent Doctorate	1 (5%) 1 (5%) 8 (38%) 7 (33%) 4 (19%)
Cultural background (White) English/Welsh/Scottish/Northern Irish/British (White) Irish Any other White background (Mixed/multiple ethnic groups) White and Asian	16 (77%) 2 (9%) 2 (9%) 1 (5%)

*One participant was pregnant at the time of interview and had a child under two years old.

Seventeen women self-reported that they had experienced mental health problems (including anxiety and depression) and/or suicidal thoughts during their current or most recent pregnancy and/or after the birth of their baby. Of these 17 women, five had not experienced any mental health problems and/or suicidal thoughts prior to their pregnancy. Thirteen women reported that they had experienced mental health problems and/or suicidal thoughts prior to their most recent pregnancy. Of these 13, 12 women also experienced poor mental health during the perinatal period, and only one participant did not. Over two-thirds of the women reported that they had received treatment and/or support for their mental health problems, but it is unclear when this was accessed (e.g., it could have been at a time prior to pregnancy and/or during the perinatal period).

### 3.2 Main findings

In the following sections, key themes related to each of the TFA constructs are discussed under the corresponding heading. Findings are presented using quantitative data from cognitive interviews, and qualitative data from semi-structured interviews. The participants thoughts regarding the response options and recall periods for the suicide-related items are also reported.

The number of positive, negative, and neutral/indifferent codes that were identified for each item using the TFA constructs as applied in this study are presented in **Supplementary Table 1**. **Supplementary Table 2** displays the number of positive, negative, and neutral/indifferent codes that were identified in relation to the response options and recall periods for each item or subscale. Both tables also present illustrative quotes per item, response option and recall period.

#### 3.2.1 Affective attitude

This construct is concerned with how women felt about the different suicide-related items. Whilst all participants expressed that it was important and necessary for healthcare professionals to ask about suicidal thoughts and/or behaviours at regular intervals throughout pregnancy and after birth, differences in how the items were worded influenced their level of comfort and reactions to them.

*Item wording and content*.

Many women were uncomfortable with items that used words such as ‘dead’ (PHQ-9, item-9; PDSS, item-7) ‘death’ (PDSS, item-14) or ‘kill’ (BDI, item-9). These words felt upsetting and confronting to them because they emphasised the finality of what thinking about suicide might result in.

“I personally do find that one a bit more of a confronting question, even just with it being ‘dead’ at the end of the sentence, that’s quite a triggering word I think for someone who maybe does have suicidal ideation, yeah, that’s a bit more of an uncomfortable question, ‘cos ‘dead’ that’s the final bit isn’t it, there’s nothing after that.” (p8).

Instead, women preferred the phrase ‘ending your life’ because this language felt slightly softer, and it focussed more on the process of suicide rather than the outcome (SRQ-20, item-17). By phrasing the item in this way, women said they would feel less defensive and more likely to engage in an honest conversation about how they were feeling.

“I’d receive this [SRQ-20, item-17] much better, if someone said to me ‘has the thought of ending your life been on your mind’ I’d be like ‘well no, it hasn’t been on my mind’ but I think if it had been on my mind, I think I’d probably be more inclined to say ‘yes’, I think it probably would open up a conversation because it’s more gentle, and in a way, it just feels less intrusive.” (p7).

Women were also more comfortable with items that asked about thoughts of ‘harming yourself’ (EPDS, item-10) or ‘hurting yourself’ (IDAS, item-15) because these terms felt more open to interpretation. As such, the women did not feel like they would be admitting to something specific if they endorsed these items, which was less frightening. However, an important caveat to this was that nearly all women interpreted ‘harming or hurting’ as non-suicidal self-harm.

“For me, that would be quite specific for self-harming, you know, even if I was suicidal at that point, I wouldn’t ever think that question had anything to do with me, I would be thinking ‘oh no, that’s self-harm.” (p4).

Women expressed mixed feelings about using the word ‘suicide’ (IDAS, item-7; Ultra-Short, item-4). For some women, this word seemed too direct and clinical, whereas for others it made the item easier to receive because it felt less personal. Two women said that using the word suicide was helpful to them because they might ‘talk themselves out’ of answering questions that used more indirect or ‘fluffy’ language. Likewise, items that used subjective and/or overly emotive language were largely disliked. Many women stated that the phrases ‘the only way out of this living nightmare’ (PDSS, item-14) and ‘the world would be better off without me’ (IDAS, item-43) were unnecessarily dramatic. Not only was this language viewed as unsuitable, but women also felt that it might not necessarily describe their experiences. As one woman said:

“I don’t like the term ‘living nightmare’, that feels like putting words in someone’s mouth in a very extreme way, you know, even if I was feeling suicidal, I would be tempted to disagree with that one because, calling it a ‘living nightmare’ might not necessarily describe my experiences.” (p3).

Some women also felt that using words such as ‘wanted to’ (PDSS, item-21, and item-35) or ‘on purpose/purposely’ (IDAS, item-9, and item-41) in relation to suicidal and/or harming thoughts and behaviours was inappropriate. They suggested that these words may reinforce feelings of shame and indirectly place a negative onus onto the woman for engaging in harming behaviours. One woman further highlighted how the use of ‘purposely’ reflects a lack of understanding regarding what living with harming thoughts and/or behaviours might look like:

“Purposely’ gives it an assumption that you know what you’re doing, and when you’re in a decline of mental health, you don’t really know what you’re doing, it makes it sound like there’s been thought behind it, but when I did it [harming myself], it was always done in a frantic, very ‘on the spot’ moment, never from a ‘I’m thinking about doing this, I’m going to go and set it up to do that, I’m now going to hurt myself’ so yeah, I think ‘purposely’ gives a really big assumption and it also detracts away from what actually an illness is like, you don’t do things ‘purposely’, you don’t even realise that you’re doing it until the act is done, and then you get all the shame and things like that afterwards, you don’t have control over it.” (p9).

#### 3.2.2 Burden and intervention coherence

This construct is concerned with how much cognitive effort was required for the women to understand and/or complete each item, and the extent to which this may affect their views regarding how the item worked to identify suicidal thoughts and/or behaviours. Overall, the clarity of items appeared to be problematic. This related to both the basic structure of sentences and comprehending the implicit meaning of concepts and phrases. Many women said that these issues might cause confusion and difficulty when attempting to answer the items because they would not understand what was being asked of them.

*Ambiguity of terms and phrases*.

Women found it easier to understand short and direct items, rather than those which contained abstract and/or complex terms. Women suggested that it was important to use plain English, and correct grammar to ensure comprehensibility, particularly for those who may not speak English as a first language and/or struggle with literacy. Many women commented positively on the relatability of items that used more ‘everyday’ language, however they also felt that sometimes these were unclear for identifying suicidal thoughts. For example, women found the phrase ‘leave this world’ (PDSS, item-35) ambiguous. Some suggested that this could mean leaving their current situation or home environment, or simply needing a break, and that they would not know how to respond to this item.

“This feels a bit more like esoteric, it feels too subjective, like ‘what does that mean [?]’, is it in like, abandon family, leave [hometown] like, or is it just voicing views on like ‘I’ve found it really hard being a mum, I found it really hard entering into this new world of parenthood’, or does it mean like, ‘I want to commit suicide’ [?], I mean most people will probably be like ‘I’m not really sure what that means’ so I don’t know if you’d ‘disagree or agree’ because I’m not really sure what this is actually getting at.” (p21).

Likewise, many women found words in the item content such as ‘often’ (Ultra-Short, item-4), ‘occurred to you’ (Ultra-Short, item-4; EPDS, item-10), and ‘on your mind’ (SRQ-20, item-17) too broad and subjective. One woman commented:

“Occurred to you often’ [?], that’s really gonna limit peoples answers because you’re gonna have somebody who thinks ‘well how often is often, how much is often, what is often [?]’ you know, is that once a week out of the past month, every single day, or just once [?], for me, just once is enough, but it couldn’t be considered to be ‘often’ in this context, so I would just get rid of ‘often.” (p13).

*Concept definitions and the implications of nuance*.

Many women struggled to understand the definitions of ‘hurt myself’ (IDAS item-9 and item-15; PHQ-9, item-9; PDSS, item-21) or ‘harm myself’ (EPDS, item-10). Most women interpreted ‘hurting or harming myself’ to mean non-suicidal harm, but some were unsure as to whether this encompassed both physical and/or psychological harm, intentional or unintentional harm, and to what degree. Women felt that these terms needed far more clarity.

“I mean ‘harming’ can be a complete range of different methods to different people, I think that’s quite broad, it could be emotional, physical, and I would think that the emotional and the physical would be treated differently in terms of ‘harm’, so I think there could be some struggle to answer that question in terms of how people interpret it … the stereotypical-ness of it is that you probably think immediately of the physical, of actually doing something to your body externally as opposed to turning to alcohol, turning to drugs, so I think it would be better if it was more granular in what it was asking for, I think it needs to be probably written out directly, of what the different things could be.” (p9).

Women had mixed feelings about whether suicidal and non-suicidal harm should be enquired about in the same item. Some women suggested that having a broad item about ‘harming’ or ‘hurting’ oneself may capture more people in need of support, whereas others saw ‘suicidality’ and ‘harming’ as two distinct phenomena that should be asked about independently. Likewise, whilst women said that it was important to identify and distinguish between a desire to die versus intentionally ending your own life, several women felt that the compound item ‘have you had thoughts that you would be better off dead or of hurting yourself in some way’ (PHQ-9, item-9) should be separated. They felt that this item was potentially asking too much e.g., passive and active suicidal ideation, non-suicidal self-harm, or a combination, which could be confusing and make it difficult to answer. A further implication was how a positive endorsement to this item might be interpreted. Some women highlighted that they may have had thoughts relating to one part of the item and not the other, and might not feel comfortable about being associated with both aspects.

“I feel like it’s better to ask them as two separate questions actually because I think the detail is good to have, whether it’s one or the other, and some people when reading this question might think ‘oh, well I don’t think I’m better off dead, but I have thought about hurting myself in some way’ and so they might kind of dismiss it or not answer it, so yeah, I think it’s better to separate them out because they might feel strongly about not answering one of them in that way and that might steer them to give a less accurate answer of how they’re feeling.” (p6).

#### 3.2.3 Opportunity costs, self-efficacy, and ethicality

This construct is concerned with how confident the women were about answering the different items (and/or feeling confident to answer them honestly) in light of their own value systems, motivation and the potential costs/benefits of their responses. Overarchingly, women talked about the stigma associated with suicidality and their fears concerning the consequences of disclosure.

*Stigma, shame, and judgement*.

For many women the concept of suicide had very negative connotations. Women expressed that the stigma associated with suicide might create a barrier to them answering these items, and/or answering them honestly and they were afraid of the consequences of endorsement (e.g., interventions from social services and/or having their baby taken away). This was particularly heightened for items that included the word ‘suicide’ or ‘committing suicide’ (IDAS, item-7; Ultra-Short, item-4), ‘dead’ (PHQ-9, item-9; PDSS, item-7), ‘death’ (PDSS, item-14) or ‘kill’ (BDI, item-9).

“That’s a really big thing to disclose to anyone, and especially if you are pregnant or if you’ve just had a baby, like your first thought would be ‘are they gonna take my baby away from me, does this mean I’m gonna end up having lots of interventions’, I would be like ‘is it safe for me to answer this question or is it going to open pandora’s box, is the help gonna be helpful [?]’… but yeah, I think that for me, the biggest barrier would be thinking ‘does this mean that social services are gonna get involved’ and that would terrify me, so that would really put me off if I’m being honest.” (p10).

Other women further highlighted the implications of using the word suicide in terms of religion and cultural differences.

“Suicide’ has got such a negative connotation, especially if you’re religious, suicide is a word that like basically, if you do it, you are super bad, you go to hell and everything else, so yeah, it’s one of these judgemental words that can make people feel taken aback from telling the truth and being honest about the answers”. (p2).

Women also talked about how the experience of suicidality stood against the traditional narratives of motherhood. This inner conflict created feelings of shame and guilt. As such, women may not want to answer suicide-related items through a fear of being perceived and/or judged as a bad mother.

“When you have like a child, there’s that feeling of guilt, you feel bad about what’s going on and you don’t necessarily want to admit that you’re feeling down or you’re struggling because it’s meant to be like the best time ever … and I think there’s that conflict there of feeling like you should be feeling great and you’ve got this amazing joy in your life and everyone says ‘oh, just enjoy every moment’ and all of that kind of stuff … so it kind of just goes against the whole narrative around having a baby and what it’s meant to be like … it would be hard to like properly admit that or answer that honestly because of all the wider conflicts as like a new mum.” (p7).

*The importance of context and framing*.

Some women found broader items (e.g., ‘the thought of harming myself has occurred to me’, EPDS, item-10) less frightening to be asked, they felt more confident in their ability to answer these honestly and suggested that these types of items might be useful for ‘opening up a conversation’. The women also talked about the importance of context, framing and normalising suicide-related items, and providing more information about why these questions were being asked as this would help to facilitate a more honest dialogue with healthcare professionals.

“You need a very sensitive introduction to a question like this, or to a whole questionnaire maybe, something like ‘we know that new parents can have thoughts about harming themselves, we want to find out if this is something that you’ve experienced so that we can put in place the right support for you’, I think you are more likely to kind of take it in if someone says it to you … normalising the fact that these thoughts can occur for new parents is important.” (p3).

#### 3.2.4 Perceived effectiveness

This construct is concerned with the extent to which women perceived the items as likely to serve their purpose. It is important to acknowledge that the ‘effectiveness’ of these items for identifying suicidal thoughts and/or behaviours is profoundly embedded within and influenced by the other TFA constructs, and heavily dependent upon additional factors such as context and individual differences. Bearing these significant implications in mind, the women did offer insights regarding the potential usefulness or relevance of some items for identifying suicidal thoughts and/or behaviours.

Many women felt that items related to ‘harming or hurting yourself’ were unclear and may not be useful or relevant for identifying suicidal thoughts as they would associate them more with self-harm. Likewise, items that used vague and/or abstract terms were not perceived to be particularly helpful, and nearly all women felt that the item ‘I thought about my own death’ (IDAS, item-14) would be ineffective for asking about suicidality. As one woman said:

“So, that doesn’t speak to me necessarily about suicide, you know, ‘I thought about my own death’ is thinking about when you might die, what might happen when you die, and if you’re pregnant or have just had children, then you may well think quite a lot about your own death, so yeah, I don’t think that would pick up on suicide at all.” (p17).

Despite the directness and heightened negative undertone of terms such as ‘suicide’ or ‘dead’, some women did feel that these words were more likely to be effective for identifying suicidal thoughts, because it was clearer what the item was asking.

“I think it’s clear and direct [IDAS, item-7], which I think if you’re trying to identify women that are likely or thinking of suicide, that’s quite a good one.” (p20).

Likewise, women saw potential utility in the phrase ‘ending your life’ (SRQ-20, item-17) for identifying suicidality because they felt that it was very specific, and it would capture the more active suicidal thoughts as opposed to passive thoughts about not wanting to be here anymore.

“This one is better than the previous ones because its ‘ending your life’, the language is softer, and it’s more direct, so with ‘ending your life’, the implication is that you would do it rather than just, you know, you not being around anymore.” (p13).

A few women also commented on the issue of interpreting items for those who don’t speak English as a first language and suggested that the word ‘suicide’ may not be as easily translatable as other terms, which may result in women from this demographic being missed.

“I’m also just thinking as well about mums for whom English isn’t their first language, and actually, ‘death’ and ‘dying’ are words that we probably come across a little bit more often, and so someone who isn’t completely fluent in English might be more likely to understand a question that’s asking about ‘death and dying’, rather than ‘suicide’ as I don’t know how ‘suicide’ translates to other languages.” (p3).

#### 3.2.5 Response options

Response options for the items varied, and women were asked to comment on their appropriateness and comprehensibility in relation to the item. Broadly, women preferred a frequency-based scale (e.g., ‘never’ to ‘every day’), or a dichotomous response choice (e.g., ‘yes’ or ‘no’) to an agreement scale (e.g., ‘strongly disagree’ to ‘strongly agree’) or severity-based scale (e.g., ‘not at all’ to ‘extremely’). However, the women also felt that it was important and necessary to follow-up all responses other than ‘no’, ‘never’, ‘strongly disagree’ and so on with a more in-depth conversation.

Many women felt that frequency-based response options were appropriate for answering difficult and sensitive items because these offered scope for disclosing some amount of thoughts based on individual comfort levels.

“I think having a scale is good, because it allows people to not commit fully ‘cos if you can like almost say ‘a bit’ then people feel a bit safer saying that ‘cos they’re not saying ‘I’ve definitely felt it all the time, but it’s just like ‘a bit.” (p1).

However, some women expressed that being asked to rate suicidal thoughts on a scale was inappropriate for identifying highly personal and distressing feelings, because this felt like a ‘tick box’ exercise. They explained how ‘yes or no’ response options were more validating of their experiences (SRQ-20, item-17; Ultra-Short, item-4).

“These would be better off as ‘yes or no’, because you’re making someone feel valid in that really strong and upsetting and difficult feeling they’re having, asking them to rate it is very odd way of approaching it I think.” (p17).

Several women also struggled to differentiate between response options such as ‘hardly ever’ and ‘sometimes’ (EPDS, item-10) because these terms were too subjective and ambiguous. Likewise, whilst some women liked the clarity of being specifically asked how many days they had experienced suicidal thoughts (PHQ-9, item-9), others felt that it was challenging for perinatal women to quantify how often these thoughts had occurred.

“I don’t really respond well to these, having to specifically pin-point it to a day, because when you are in those kind of head spaces, and especially having like a baby and stuff, when you’re tired, you’re not counting days, it’s either going to be really frequent or it’s not, yeah I don’t think you’re gonna be counting to see if it’s ‘half the days’ or not.” (p8).

Most women had strong negative reactions to the agreement-type and severity-based response options. Women said that using the terms ‘agree or disagree’ (PDSS, subscale) felt like a work performance review or generic questionnaire and was highly inappropriate for such a sensitive topic. They also stated that there was no benefit to having the option of ‘neither disagree or agree’, nor did they understand how to differentiate between ‘agree’ and ‘strongly agree’. Similarly, the women found the severity-based options (IDAS, subscale) very confusing, and not fitting at all for the types of questions that were being asked.

“I mean, ‘I had thoughts of suicide, a little bit’ I just can’t imagine what thoughts of suicide ‘a little bit’ are [?], the rating thing isn’t any good I don’t think, and ‘extremely’, something about that doesn’t sit well either, I mean apart from the fact that it doesn’t make sense, I think if I was sitting down filling in a questionnaire, and I was feeling extremely suicidal, it just wouldn’t feel comfortable to me that wording.” (p17).

Women had mixed feelings about the statement-based response choices (BDI, item-9). Despite all women disliking the content of this item (e.g., ‘killing myself’), some did see value in the format because it provided a distinction between passive versus active thoughts and intent. Other women said that this style included too much information to process, which would make them feel overwhelmed.

#### 3.2.6 Recall period

The recall period for the items ranged from ‘the past seven-days’ to ‘the past month’. Women were asked to provide their thoughts on these timeframes to explore appropriateness and relevance to the items. Overall, the women felt that the recall period for suicide-related screening items should be longer rather than shorter, although some did comment that shorter recall periods may be necessary and/or useful in certain instances (e.g., to monitor symptom changes for those in specialist care).

The women’s preference for wider timeframes was largely due to an overarching concern that people may be missed if the recall period was limited to seven-days or two-weeks. Several women talked about the fluctuating nature of suicidal thoughts and highlighted that these may vary significantly from one day to another. As such, women felt that some suicidal thoughts might not be captured by a shorter timeframe because people may interpret this literally and therefore not disclose anything that had happened outside of the specified period.

“You might think, ‘well, at the start of the month, I was not in a good place, but this week I feel better’ so then, you know, you could answer ‘never’, because it’s asking about the past seven-days and you’re just sort of like getting missed, especially someone like me, I do sort of have these like episodes, where I’ll be well for a few weeks, and then I can sort of like dip again, so very specifically seven-days I could answer ‘never’, so it’s tricky really.” (p14).

Some women also expressed that putting a time restriction on these types of questions could minimise and devalue their experience of suicidal thoughts. These women said that they might assume that suicidal thoughts were not important to maternity caregivers unless they had occurred within the specified timeframe, which may then deter them from disclosing sensitive and difficult information in case it was dismissed.

“I actually think it should be over a longer period because in a way it’s quite dismissive isn’t it, if I had thought about harming myself, I don’t know whether I’d feel a bit like ‘oh maybe I shouldn’t say anything’ or I might feel like ‘oh maybe it doesn’t matter if it was over seven-days ago ‘cos it’s not as important.” (p7).

Lastly, whilst most women thought that applying some type of timeframe was necessary when asking about suicidal thoughts and/or behaviours, they also felt that this needed to be contextualised to their current situation. Several women suggested that a more appropriate framing of these items might be to ask ‘since pregnancy or since your baby was born, have you…’ because this would provide both a suitable recall period and it would situate the items in terms of being a pregnant woman or new mother.

“Rather than have it kind of in a timeframe, it almost needs to be like ‘since you’ve been pregnant or since birth’, do you know what I mean [?], ‘cos it’s more about your experience of the pregnancy itself than it is about the time, so I’m wondering that for all the questions really.” (p21).

## 4 Discussion

This study assessed the acceptability and content validity of 16 suicide-related items with pregnant and postnatal women using the TFA, and two additional categories of ‘response options’ and ‘recall period’. It also explored the barriers and facilitators that affected women’s reactions and responses to these items. This is the first time that the acceptability of different suicide-related items has been examined in perinatal populations, and the first time that the TFA has been applied in this context. Novel findings of this research are that the suicide-related items assessed in this study were largely unacceptable to pregnant and postnatal women. This finding is contrary to research that suggests more general perinatal mental health screening and/or common screening measures are acceptable to perinatal women (37–39). Therefore, this study makes an important contribution to the literature in the following ways: (i) it offers insight into how perinatal women feel about different suicide-related items that are embedded within measures that screen more widely for depression and/or other mental health problems (including their response options and recall periods); (ii) it highlights factors that may facilitate or prevent perinatal women from answering suicide-related items, and/or answering them honestly; and (iii) it provides a nuanced understanding regarding the utility and appropriateness of using these screening items to identify suicidal ideation and/or suicidal behaviours in perinatal women, which may be valuable and relevant in both research and clinical settings.

The TFA provided a structured and meaningful approach for exploring the acceptability of suicide-related items with perinatal women. Some of the TFA constructs were collapsed in this study to create a coding framework that was suitable for assessing the participant data in line with the study design and research aims (see **Table 1**). Clustering the components in this way allowed for greater depth in understanding how different aspects of acceptability affected the content validity of items, and it provided scope for identifying barriers and facilitators to disclosure when applying these items in the ‘real world’ context. The results suggested that word choice and the terms used to characterise suicide affected women’s comfort with and comprehension of the items, and their willingness to engage.

In the following sections, the findings related to the TFA constructs for each suicide-related item or subscale have been summarised to provide an overall assessment of their acceptability. The corresponding response options and recall periods are discussed in terms of their key strengths and weaknesses. The clinical implications of applying these measures in practice is also considered.

*SRQ-20, item 17*.

Overall, women were most comfortable with SRQ-20, item-17, which asks ‘has the thought of ending your life been on your mind’. Women said that the phrase ‘ending your life’ felt slightly softer than other terms and made the item less confronting to answer honestly. Women also saw potential utility in this item and its response options (‘yes/no’) for identifying suicidal thoughts in perinatal women, although some felt that the latter part (‘on your mind’) was confusing and should be modified or removed.

*EPDS, item-10*.

Similarly, women generally found EPDS, item-10, less upsetting than other items. However, many interpreted ‘harming myself’ to mean non-suicidal self-harm, and did not think the item was specific enough for identifying suicidal thoughts. This finding is in line with previous research that also highlighted the ambiguity of this item (37, 55). Furthermore, whilst most women felt that frequency-based response options were appropriate, some struggled to differentiate between ‘hardly ever’ and ‘sometimes’, which created additional confusion in answering the item accurately.

*PHQ-9, item-9*.

Women had mixed feelings about PHQ-9, item-9. Some women thought that asking about suicide and self-harm in one item was useful, whereas others felt that these needed to be addressed separately. Several women said they would not know how to answer this item, and/or would not feel comfortable doing so because it was attempting to ask too much. Likewise, the phrase ‘better off dead’ was largely disliked. Women found this distressing and said that it may prevent them from answering the item. Previous research has observed similar hesitancy from perinatal women towards PHQ-9, item-9 (53, 54), but it was not clear whether this related to the specific item wording or to the topic of suicide more broadly. Differentiating the terms ‘several days’ and ‘more than half the days’ and having to quantify their answers was problematic.

*PDSS, suicidal thoughts subscale*.

The PDSS suicidal thoughts subscale comprised five items. Generally, women found these items to be overly emotive or dramatic, and/or too abstract for identifying suicidal thoughts in perinatal women. Women also thought that the agreement response option, was unsuitable because it felt generic and undermined the seriousness of the topic. They further struggled to understand what determined ‘strongly disagree’ from ‘disagree’ or ‘strongly agree’ from ‘agree’ and did not see value in the option of ‘neither disagree or agree’ for items related to suicide and/or self-harm.

*IDAS, suicidality subscale*.

The IDAS suicidality subscale comprised six items. Asides from item-43 (‘the world would be better off without me’) and item-14 (‘I thought about my own death’), women found these items relatively direct and clear. However, most women felt that three of these items (item-9, item-15, and item-41) were self-harm specific, and unlikely to be effective at identifying suicidal thoughts. Likewise, all women were uncomfortable with item-41 ‘I cut or burned myself on purpose’ and felt that this type of question should only be asked as part of a comprehensive assessment, and not used for screening. Women also largely disliked the severity scale response options. For example, the response option of ‘extremely’ in answer to item-7 ‘I had thoughts of suicide’ did not appear to make sense, nor was it befitting to the item.

*BDI, item-9 and the Ultra-Short Maternal Mental Health Screen, item-4*.

Women had the strongest negative reactions to BDI, item-9, and item-4 from the Ultra-Short, which use the terms ‘killing myself’ and ‘committing suicide’, correspondingly. These items also elicited the most concern from women in terms of perpetuating stigma and inciting fear about the consequences of disclosure. Nearly all women felt that these items were inappropriate for identifying suicidality in perinatal women and they would not want to answer them.

*Recall period*.

Recall periods for the items ranged from ‘seven-days’ to the ‘past month’. Many women felt that longer timeframes should be applied to these items to avoid cases being missed, prevent women’s experiences being minimised and to contextualise these items around being a pregnant or postnatal woman. Several women suggested using the phrase ‘since pregnancy’ or ‘since the birth of your baby’ (or similar) as they found this more relatable to their current situation, and easier to reflect upon in terms of when and/or how frequently thoughts were happening.

### 4.1 Implications for practice and future research

Many suicide-related items that are embedded into existing screening measures for depression and/or other mental health problems may be unacceptable to perinatal women in their current form. Identifying pregnant or postnatal women who may be experiencing suicidal ideation and/or suicidal behaviours poses several challenges in the maternity care context. Pressures due to financial constraints, a lack of resources, staff shortages and increasing demands and expectations are common across health services. Maternity care practitioners often have little time during routine appointments to enquire about mental health problems with perinatal women, which further increases the potential for cases being missed. Whilst common mental health problems are not universally screened for during pregnancy and after birth in the UK, screening measures do offer a pragmatic approach for identifying women who may require additional support. These measures are generally brief and easy to administer, with a relatively small burden upon resources. However, healthcare professionals and researchers should be aware of the limitations of using the measures assessed in this study to identify possible suicidality in pregnant or postnatal women because the content, comprehensibility and appropriateness of these items and their corresponding response options and recall periods may create and/or reinforce barriers to women’s disclosure. There may be some value in using screening measures to identify the presence or absence of suicidal ideation in perinatal women as part of a stepped approach for identifying those who may require additional support, but the development of specific measures that are acceptable to perinatal women for this purpose is warranted. Such measures should not be used to assess suicide risk, predict future behaviours and/or determine treatment outcomes, but instead to indicate that further comprehensive psychosocial assessment might be necessary. New measures should be developed in accordance with evidence-based guidelines such as the Consensus-based Standards for the Selection of Health Measurement Instruments (COSMIN) (74) and in collaboration with perinatal women. It is also important for maternity care practitioners to consider the framing of suicide and/or self-harm questions when engaging in discussions with perinatal women because wider evidence suggests that subtle differences in wording and communication style can influence and create additional barriers to disclosure (75–77). Adopting a sensitive, open, and non-judgemental approach may help to foster a trusting and safer environment for perinatal women to share their thoughts and feelings.

Furthermore, research is needed to explore perinatal women’s experiences and views on the broader implications of discussing suicide in maternity care settings. This is important because many women commented on the influence of context upon their willingness to disclose suicidal ideation and/or behaviours. Factors including how suicide-related screening questions might be administered, when and/or how frequently they might be asked, and who should ask them, continuity of care, referrals to specialist services and general knowledge about perinatal mental health conditions may be relevant for developing appropriate and acceptable approaches for identifying suicidality in pregnant or postnatal women. Likewise, cultural differences, religious beliefs, language and translation issues, and social factors may create unique challenges for identifying women from different ethnic and minority groups, and future research should explore the wider barriers and facilitators, and the acceptability of suicide-related measures with women from more diverse backgrounds. It would also be useful to examine the acceptability of suicide-related items with pregnant and postnatal women in treatment settings as their views may differ from those identified in non-clinical samples.

### 4.2 Strengths and limitations

Using a theoretical framework to explore the acceptability of suicide-related items with perinatal women is an important strength of this research. Compared to more general approaches for assessing acceptability, the TFA provided a systematic and comprehensive approach for informing the study design and materials, and analysing the in-depth interview data, which enabled key components of acceptability to be explored. Furthermore, given that suicidal ideation and/or behaviours are still highly stigmatised, and that recruiting pregnant women or women with a new baby poses unique challenges, the sample size and inclusion of participants from across the UK were also significant strengths of this study.

Several limitations of this study also need to be acknowledged. Firstly, using the TFA to assess the acceptability of different suicide-related items was a novel approach. The TFA was originally developed to assess the acceptability of healthcare interventions rather than measurement scales per se, hence there was a scarcity of literature or clear guidelines for how the TFA could be applied in this context. In this study, some of the TFA constructs were clustered to create an appropriate framework for exploring the data in line with the research aims. This may have affected the findings because not all constructs were assessed as unique and independent components of acceptability. Likewise, previous research has reported overlap and/or ambiguity differentiating some of the TFA constructs in certain contexts (58, 61), and similar interrelatedness was identified here. This was particularly evident for ‘perceived effectiveness’ which was heavily embedded within and influenced by the other TFA constructs. Therefore, the utility of ‘perceived effectiveness’ as a distinct component for assessing the acceptability of screening measures may be limited, and more suited to healthcare interventions with clearer behavioural and/or treatment outcomes. Secondly, the suicide-related items used in this study were taken from several wider measures of depression, anxiety and/or psychological distress. Examining these specific items in isolation to the full measurement instrument(s), and outside of their intended context, may also be a limitation of this research. The utility of these items when asked in conjunction with their counterpart items may be more acceptable and relevant for identifying wider mental health problems in perinatal women. Likewise, items from the PDSS suicidal thoughts subscale (five items) and the IDAS suicidality subscale (six items) were assessed as standalone items and not in combination together for identifying suicidality. Therefore, the relevance, comprehensibility, acceptability, and appropriateness of some of these items may have been affected. Similarly, women were not asked to directly comment on the use of depression measures to screen for suicidal ideation and/or behaviours, so the broader acceptability of this approach cannot be inferred. Lastly, the sample comprised mainly White British pregnant and postnatal women with a high level of education. As such, the views of women from more diverse ethnic, cultural, social, and religious backgrounds is required.

### 4.3 Conclusions

This study assessed the acceptability and content validity of suicide-related screening items with pregnant and postnatal women using the TFA. Whilst all participants agreed that it was important and necessary to ask about suicidal thoughts and behaviours during the perinatal period, the findings from this research suggest that many existing suicide-related items that are embedded into wider measures of depression and/or other common mental health problems are unacceptable to perinatal women in their current form. Item-17 from the SRQ-20 may have some utility for identifying suicidal ideation in clinical and research settings, although modifications to the latter part of the item (‘on your mind’) should be considered. Maternity practitioners and researchers need to be cautious about using the measures explored in this study for identifying suicidal ideation and/or behaviours in perinatal women because their content, comprehensibility, and appropriateness may create and/or reinforce barriers to disclosure. Stigma, perceived judgement, and a fear of the consequences of disclosure are significant factors that may prevent women from being honest about how they are feeling. More research is needed to explore the acceptability of discussing suicidality in maternity care settings, and the development of specific screening measures for identifying suicidal ideation in perinatal women are warranted. Such measures may help to facilitate the early identification of those who may require additional assessment and support, which may lead to better outcomes for women, their children, and families.

## Data availability statement

The datasets generated and analysed during the current study are not publicly available due to the sensitive nature of the data. Requests to access the datasets should be directed to Elizabeth Dudeney, elizabeth.dudeney@city.ac.uk.

## Ethics statement

The studies involving humans were approved by School of Health and Psychological Sciences Research and Ethics Committee (SHPS REC) at City, University of London (reference number, ETH2122-0757). The studies were conducted in accordance with the local legislation and institutional requirements. The participants provided their written informed consent to participate in this study prior to their interview, and they had the opportunity to withdraw from the study at any time up until the point of data analysis, without needing to provide a reason.

## Author contributions

ED: Conceptualization, Data curation, Formal analysis, Investigation, Methodology, Writing – original draft. RC: Conceptualization, Formal analysis, Supervision, Writing – review & editing. SA: Conceptualization, Supervision, Writing – review & editing. RM: Supervision, Writing – review & editing.
