# Supplementary material for: Acceptability and content validity of suicidality screening items: a qualitative study with perinatal women
Source: Front Psychiatry. 2024 Apr 11;15:1359076. doi: 10.3389/fpsyt.2024.1359076 (PMC11044181; doi:10.3389/fpsyt.2024.1359076)
Supplement: Supplementary file 1 [file Table_1.docx]

**Supplementary Table 1**

Number of positive, negative, and neutral/indifferent TFA construct coding instances for the suicide-related items, with illustrative participant quotes

TFA construct

| Measure | Affective Attitude | Burden & intervention coherence | Opportunity costs,  self-efficacy & ethicality | Perceived effectiveness |  |
| --- | --- | --- | --- | --- | --- |
| BDI, item-9* | | (+) = 6  *“It’s quite interesting to have the second choice because I don’t think that’s sort of been mentioned, like they’ve said ‘suicidal thoughts’ but there has not been the kind of ‘but I wouldn’t actually do it’ which some people might feel more comfortable saying, like ‘I don’t want to say I’ve had suicidal thoughts without saying that I wouldn’t actually do it” (p1)*  **(-) = 29**  *“It’s very strong language, it even like made me triggered a bit even though I don’t have these thoughts because, it’s very, very strong for me, I would definitely be taken aback to answer this, like I found it scary as a typology of questions, ‘kill myself’ is very aggressive and it sounds very twisted, yeah, to put it bluntly, I really didn’t like this question” (p2)*  (+/-) = 0  *N/A* | (+) = 5  *“This seems quite straight forward really, it kind of distinguishes between not having any suicidality, to having thoughts but not a plan, to having a plan, to being actively suicidal, yeah, this seems the most clear so far actually, yeah kind of seems to distinguish and solve a lot of the discussions we’ve had before” (p5)*  **(-) = 12**  *“When you have people who struggle reading questions, perhaps their English isn’t to a stage they can pick up that much information, that is a lot to read first of all, that’s a lot to take on board, and then, not only do you have to read each of the things, you’ve then got to read it several times before you can answer like ‘which category do I fall into’, I think if you’re not uneducated, or if you had a woman who struggled with English at school, or if you had someone with English as a foreign language, those more vulnerable people, I think they would struggle to answer it, ‘cos I’ve had to read it several times to then think about which one I would tick off” (p16)*  (+/-) = 0  *N/A* | (+) = 0  *N/A*  **(-) = 9**  *“This is less sort of like ‘inviting’ to open a conversation, like the question before helps you to open up [SQR-20] where you might talk a bit more about it, whereas as this one just feels like you wouldn’t necessarily, I’m thinking about if I had [had suicidal thoughts], whether I’d ever tick ‘I have had thoughts but I would not carry them out’ because then I would feel like it could ring alarm bells, I’d probably just tick ‘no’ ‘cos you’d just worry about the consequences too much” (p7)*  (+/-) = 0  *N/A* | **(+) = 5**  *“I think it’s a useful one ‘cos you’re really able to differentiate when you get that detail ‘who are the people with thoughts, who are the people who are thinking about it, and who are the people who are really on the edge’, in one question, you really get that, ‘cos you’re putting them all next to each other and they’ve got to make a choice where are they on that spectrum, so yeah, I think that’s quite a good one” (p6)*  (-) = 2  *“It seems very out of place this question, compared to all the others, like much less subtle and much less tactful, yikes, I think that there’s much better ways [to ask] and we’ve seen some of them, yeah, I just wouldn’t ask this question in this way, it’s just gonna scare people I think, or upset people, and I feel like you’re going to put people off” (p13)*  (+/-) = 0  *N/A* |
| EPDS, item-10* | | **(+) = 20**  *“It’s a pretty valid question, I don’t feel like I’m off put by anything about it” (p04)*  (-) = 6  *“[It’s] an emotive question which might make it harder to come to the reality of ‘oh yes, actually I have thought about harming myself” (p06)*  (+/-) = 0  *N/A* | (+) = 8  *“I feel like this is a fairly self-explanatory question, I think- if I was reading it, I would understand what it was getting at” (p21)*  **(-) = 15**  *“For women who don’t have that specific knowledge, this would be a bit of a tricky question, because what is ‘harming yourself [?]” (p05)*  (+/-) = 1  *“I understand what it’s asking me, it just feels a bit convoluted” (p12)* | (+) = 2  *“I feel like it’s easier to admit that it’s ‘occurred to you” (p11)*  **(-) = 9**  *“I think that for me, the biggest barrier would be thinking ‘does this mean that social services are gonna get involved’ and that would terrify me” (p10)*  (+/-) = 1  *“That really depends on the context, I think it depends on who's asking it, and it depends what situation they're in” (p21)* | (+) = 2  *“I think the term ‘harming myself’ is very general so I would interpret the question as that they’re asking about self-harm, which at the very extreme end might be suicide” (p18)*  **(-) = 12**  *“I would definitely say that I would not equate that question to suicide or suicidal thoughts, that wouldn’t come into my mind” (p10)*  (+/-) = 0  *N/A* |
| IDAS, item-7* | | **(+) = 15**  *“I think for me personally, I prefer to read a question where it’s saying ‘suicide’ as opposed to ‘death’ or ‘dead’, I think because suicide maybe in a way hides the actual outcome of what it means” (p9)*  (-) = 12  *“It’s just quite a difficult word isn’t it [suicide], and it’s not a word we use lots in kind of general language… I think if I was on the receiving end of the question and I was feeling really vulnerable, I would probably prefer something like ‘have you had thoughts of ending your life’ rather than ‘suicide” (p7)*  (+/-) = 0  N/A | **(+) = 13**  *“I like the clarity of that, I think it is really clear, you know exactly what you're answering, and it's not emotive in any way, and everyone knows what suicide means” (p17)*  (-) = 3  *“Well, the first thing that annoys me is that it’s not good English, like it’s not proper grammar, but I say that kind of in a trite way, you know, I can deal with bad grammar, but it actually means that the question isn’t easy to answer, so I think that’s an issue” (p18)*  (+/-) = 0  *N/A* | (+) = 3  *“I don't have that kind of fear or shame around it, so for me, it's fine [the question]” (p12)*  **(-) = 6**  *“It’s the intent and shame I think, you know it’s quite shameful and it used to be a crime right if you committed suicide, and I think especially when it’s religion backed, there’s a lot of issues there with the concept of suicide… so there is a lot of stigma attached, like I would feel uncomfortable if I were suicidal and for somebody to say that to me because of everything that’s associated with that” (p13)*  (+/-) = 0  *N/A* | **(+) = 11**  *“I prefer that question in some ways, and it’s direct, it definitely answers the criteria you’re looking for you know, suicide directly, yeah” (p6)*  (-) = 0  *N/A*  (+/-) = 0  *N/A* |
| IDAS, item-9* | | (+) = 8  *“It seems like a good question because it’s very accepting with that word ‘purposely’, it actually seems to resolve some of the issues I raised about the previous one, about self-harm and intent” (p5)*  **(-) = 10**  *“I don’t think there’s any care in that question either… I feel like it’s almost like a tick-box exercise, like they need to figure out whether I do feel like that, and then if I tick such and such then- yeah, there’s no care about that, I think sometimes when you’re pregnant, you need a bit of love don’t you [?]” (p16)*  (+/-) = 1  *“There might be a difference between doing something relatively minor versus something that could lead to a potentially life-threatening injury, so you’ll probably dig a bit under the surface with these, but you won’t get everything you wanted” (p10)* | (+) = 8  *“It does make sense and it's very clear to me what you're asking me, you know ‘have you actually done it, are you doing it’ rather than ‘have you had feelings of doing it’, it’s ‘I've actually done it’, it's relatively easy” (p17)*  **(-) = 16**  *“The only thing that I would say that is, somebody might not be hurting themselves on purpose right at this time, but they could have in the past, which means they are then at risk of sort of doing it again, so ‘I have or I do hurt myself’ I think would probably be better, because like today I would answer that as ‘not at all’, but I have previously, so therefore it's on my mind that perhaps I might one day do that again” (p14)*  (+/-) = 0  *N/A* | (+) = 1  *“I actually think that it doesn’t have any sort of judgement or negative connotations” (p2)*  **(-) = 3**  *“I think that someone would be really ashamed to say, ‘I hurt myself purposely’ I mean, I certainly wouldn't want to admit it, it's a horrible thing to say isn't it ‘I hurt myself purposely’, it’s horrible, yeah, it’s awful, it's really sad” (p15)*  (+/-) = 0  *N/A* | (+) = 0  *N/A*  **(-) = 8**  *“It can capture a lot of behaviours, but it doesn't tell you about suicide I don't think, and it wouldn't be necessarily that helpful understanding whether someone feels suicidal” (p21)*  (+/-) = 0  *N/A* |
| IDAS, item-14* | | (+) = 6  *“It’s almost a little bit more abstract, so it makes you feel like a lot more comfortable taking about it and opening up” (p2)*  **(-) = 8**  *“I don't think this question is very helpful because it means so many different things to different people, it's really difficult to answer that one, when I first saw ‘death’ I thought, ‘oh my gosh, that's really harsh’, it's a hard word to say, isn't it” (p15)*  (+/-) = 1  *“It doesn’t feel like they’re [this subscale] trying to like dress it up with any different language, it’s just factual” (p10)* | (+) = 2  *“I mean, again, that question’s clear and easy to answer” (p18)*  **(-) = 18**  *“It’s quite passive, so not ‘I thought about killing myself’ but just ‘thought about my own death’, so is that in the sense of how it would happen, or how would I do it [?], or more along the lines of how will I die as in ‘will I get old, will I get hit by a car, will I get cancer [?]’, so I don’t know, I don’t feel like it’s specific enough because sometimes I do [think about death] and it makes me worried, but it’s not because I’m suicidal, it’s the opposite of that, like I don’t want to die, and I think about it and it scares me, so it’s definitely not specific enough” (p13)*  (+/-) = 0  *N/A* | (+) = 0  *N/A*  (-) = 0  *N/A*  (+/-) = 0  *N/A* | (+) = 1  *“I think it still gets to the point, and I think it's still relevant” (p12)*  **(-) = 17**  *“That one’s pretty vague for me, I wouldn’t necessarily correlate that with suicide, thinking about your own death could be an accident, it could just be worrying about who would look after your kid afterwards, I mean yeah, I’ve thought about my own death loads and life insurance, that kind of stuff, so that question to me wouldn’t necessarily really even have anything to do with mental health, never mind suicide” (p8)*  (+/-) = 0  *N/A* |
| IDAS, item-15* | | **(+) = 9**  *“That can really help like even opening the conversation and like sliding into the topic without feeling too [judged] yeah, making people feel comfortable, and women feel comfortable because it’s less judgement when it’s just like a thought, definitely” (p2)*  (-) = 2  *“That’s maybe a little bit too soft, I can see why that might be a bit easier for some people to answer, I think it’s just my preference for being quite blunt, that [question] would kinda maybe give me a wee bit of pause on that” (p8)*  (+/-) = 1  *[Same comment as IDAS, item-14]* | (+) = 2  *“I would say that it’s very clear, you know, if you have like a collective understanding of what it means to ‘hurt’ yourself” (p10)*  **(-) = 11**  *“It’s all around that assumption of ‘well, what is the question meaning, what is it wanting me to think about here’ [?], is this question wanting to gather a bigger picture of all the different ranges of how you hurt yourself, you know, I could hurt myself in several different ways, emotionally and physically, it’s too broad and again, someone who’s probably answering this is still probably not in the right frame of mind, and I think that could cause confusion” (p9)*  (+/-) = 0  *N/A* | **(+) = 2**  *“I think there are a lot of things that people might consider as ‘hurting myself’ but wouldn’t consider ‘self-harm’, and yeah, I think with that stigma, if someone read ‘I thought about self-harming’ they might be thinking, ‘if I say that, are they gonna think I’m attention seeking, are they gonna think I’m manipulative’, all that kind of stuff, whereas ‘hurting myself’ feels less stigmatised I think” (p3).*  (-) = 0  *N/A*  (+/-) = 0  *N/A* | (+) = 0  *N/A*  **(-) = 8**  *“I think again, I wouldn’t link it to suicide necessarily, I would be thinking about self-harm, I think it would be asking about ‘have you self-harmed or thought about self-harming’, I mean we’ve spoken about the kind of ambiguity around ‘hurting and harming’ and how to me, that doesn’t mean suicide” (p11)*  (+/-) = 0  *N/A* |
| IDAS, item-41* | | (+) = 3  *“On purpose’ is important here obviously because, you know, we burn ourselves all the time” (p13)*  **(-) = 21**  *“I would rather answer a more general question and then be asked a bit more in person about what that was… also if I read that I might think ‘oh well if I haven’t cut or burned myself, I haven’t really self-harmed, I am not going to be taken seriously’ you know, if people are kind of hurting themselves or having thoughts of self-harm, but they’re not getting the support that they would like to be receiving or that they feel they need, and then they see a question like this, they might think ‘oh, if I cut or burn myself then maybe finally someone will listen to me” (p3)*  (+/-) = 1  *[Same comment as IDAS, item-14]* | **(+) = 5**  *“I guess this is just a bit more of a specific one, so yeah, it's pretty straightforward” (p20)*  (-) = 2  *“I think that’s very specific, maybe that could be an example given, like ‘I deliberately hurt myself, for example, by cutting or burning’, I think that might be clearer, ‘cos you know, you might have harmed by another method and then you’d answer ‘no” (p11)*  (+/-) = 0  *N/A* | (+) = 0  *N/A*  **(-) = 6**  *“It’s just very specific, I think that's actually one of the ones that I view as like a bit maybe too ‘on the nose’, because I think people might be quite ashamed, like people might be able to tell you like, ‘oh I self-harm occasionally’ but I think there'd probably be an element of shame around the exact action, that feels very internal, so I think that might spook people a bit” (p21)*  (+/-) = 0  *N/A* | (+) = 0  *N/A*  **(-) = 8**  *“It’s not that useful diagnostically for ‘are they hurting themselves, are they suicidal’ it probably doesn’t help in that way, so yeah, I’d probably take that question out, I can’t think of any reason to keep it in” (p6)*  (+/-) = 0  *N/A* |
| IDAS, item-43* | | (+) = 10  *“So, from my lived experience, that’s a great question and I’m glad that it’s there and it’s worded in a great way ‘cos it’s not just the ‘I don’t want to be here’ it’s also tapping into the ‘why’, so that’s a really good example from my experience of what would have worked” (p5)*  **(-) = 11**  *“It's the same as some of the other more emotive ones, like putting words in someone's mouth, and if that doesn't resonate with the way they see their suicidal thoughts, you're going to not pick up on it… it should say something about people rather than the ‘world’, so your loved ones, but yeah, the ‘world’ is so abstract and odd” (p17)*  (+/-) = 0  *N/A* | (+) = 2  *“That’s nice and clear to me” (p5)*  **(-) = 13**  *“I don’t find it very clear, the ‘world’ is far too big to make a call on that, do they mean the people in the world, do they mean environmental issues or [?], I’m not sure what you would get from that question that’s useful” (p18)*  (+/-) = 0  *N/A* | (+) = 1  *“That [question] would have opened up a dialogue with the professionals, yeah” (p5)*  **(-) = 3**  *“I do feel that it has a little bit of an issue of putting words into your mouth that you might not actually feel” (p3)*  (+/-) = 0  *N/A* | (+) = 4  *“I think that’s a really good question to have in there because that is the sort of thing that goes through your head, that’s- like your own language- that is the sort of thing that flickers through your head” (p4)*  **(-) = 8**  *“It could just be leaving- it could be packing up your stuff and going, leaving baby with the dad and going, that’s not necessarily related to suicide either, it might just be that you don’t think you have enough to offer them in terms of you don’t think you’re a good enough mum, yeah I don’t think that’s a particularly great question, or clear, I don’t think it gives enough clarity for kinda what you’re trying to ask about” (p8)*  (+/-) = 1  *“Does that tell you about suicide [?], it sort of does, if it was in the right context and being asked sensitively, I could understand that they were asking about suicide” (p21)* |
| PHQ-9, item-9* | | **(+) = 14**  *“In terms of the question, it's really good because it encompasses ‘better off dead’ as well as ‘hurting yourself’, so instead of just being hurting yourself, which for me, hurting yourself is not killing yourself, or that you'd be better off dead, I think that is a good way of phrasing it” (p15)*  (-) = 11  *“I feel like it’s better to ask them as two separate questions actually… some people might think ‘oh, well I don’t think I’m better off dead, but I have thought about hurting myself in some way’ and so they might kind of dismiss it or not answer it, so I think it’s better to separate them out because they might feel strongly about not answering one of them in that way and that might steer them to give a less accurate answer of how they’re feeling” (p6)*  (+/-) = 1  *“I guess it depends on what you're trying to get out of the question” (p20)* | **(+) = 8**  *“I think that one’s a bit clearer in terms of what the question’s asking, so it’s kind of doing both isn’t it, it’s the kind of ‘self-harming’ and ‘suicide’ potentially, but it includes both… I think I would understand what I was being asked there and be able to answer it accurately” (p11)*  **(-) = 8**  *“It’s a bit confusing, like the fact that the question has got both elements to it… like what are they looking at [?], it’s better to split these two out, then it’s really clear which one you’re responding to… it’s just a bit confusing isn’t it because what if you had a thought that you’d be better off dead once and then you thought about hurting yourself four times, like how would you answer that question [?], so you’re not necessarily gonna get the information that you’d want as a professional out of the answers to this question” (p7)*  (+/-) = 0  *N/A* | (+) = 3  *“I would be happy enough to answer that honestly” (p4)*  **(-) = 6**  *“I definitely appreciate that this not necessarily the case for everyone, but maybe some people wouldn’t want to be associated with ‘killing’ themselves just because they thought of ‘hurting’ themselves” (p13)*  (+/-) = 0  *N/A* | **(+) = 7**  *“I think the phrasing of ‘thoughts that you’d be better off dead’ has that kind of generalness to it, in that its gonna capture the people with the kind of passive suicidal ideation as well as the active, whereas ‘I wanted to harm myself’, ‘I wanted to end my life’, you wouldn’t capture the people who were having thoughts of ‘oh, I just wish I could go to sleep and not wake up’, whereas I think that would be captured with this question” (p3)*  (-) = 3  *“You're only given one option to answer both questions, so actually, you should split those up, because thinking about it, it doesn't tell you about suicide, it might tell you that someone has thought about hurting themselves, but not about actual suicide” (p21)*  (+/-) = 0  *N/A* |
| PDSS, item-7* | | (+) = 14  *“I mean it’s pretty to the point isn’t it, but I think the question needs to be asked, and I wouldn’t feel offended by being asked that” (p11)*  **(-) = 20**  *“I’d feel a bit uncomfortable about the word ‘dead’, the word ‘dead’ just feels really like direct, final, extreme” (p7)*  (+/-) = 1  *“I just would not know how someone in that situation wants to be asked that, I just can't even put myself there” (p19)* | **(+) = 8**  *“better off dead’ is much clearer in what you're asking” (p17)*  (-) = 7  *“I guess the only sort of ambiguity there is like the ‘I’ve started thinking’, you know, maybe if you’ve got a history of feeling suicidal, you might not have ‘started’ thinking it, it might have come back” (p10)*  (+/-) = 0  *N/A* | (+) = 1  *“I'd be able to be honest” (p17)*  **(-) = 9**  *“It’s very difficult to answer honestly which is my only thought, it’s so tricky as a woman, especially as a pregnant woman where you’re constantly judged from midwives, to be honest in answering this type of question because you are afraid that they are gonna take away your baby, so it could be very triggering if the answer is something other than ‘never” (p2)*  (+/-) = 0 | **(+) = 8**  *“This one would have really rung true for me at certain points, you know, ‘thinking’ that was definitely a thought I was having, so that sort of taps into it” (p5)*  (-) = 2  *“Because it is that everyday language, it feels more like when you might have a sudden flash of like ‘oh god I just wanna not wake up, I’m so tired’, you know, it's almost quite casual and we sometimes have these crazy thoughts when we're really tired, and it feels more like that than like a really serious question I suppose” (p21)*  (+/-) = 0 |
| PDSS, item-14* | | (+) = 7  *“That felt OK to me reading that one out” (p3)*  **(-) = 24**  *“I would prefer it if it didn’t have the thing about the ‘living nightmare’, ‘cos I think that’s really putting words in someone’s mouth, and that might not be their experience” (p10)*  (+/-) = 1  *“It’s quite emotive and I honestly don’t know either way if that’s a good thing or a bad thing” (p13)* | (+) = 1  *“It’s understandable in a sense ‘cos it’s in plain English and it’s almost conversational, casual language, which might make it better” (p1)*  **(-) = 4**  *“Not only is it offensive to have to rate that, but it's also really confusing because it's such a lot, it's a long complex sentence” (p17)*  (+/-) = 0  *N/A* | (+) = 0  *N/A*  **(-) = 15**  *“Framing it as like a ‘living nightmare’, even if you may be thinking that, it would be hard to kind of like properly admit that or answer that honestly because of all of the wider conflicts” (p7)*  (+/-) = 0  *N/A* | **(+) = 3**  *“I think the question is appropriate for sure in terms of the topic” (p5)*  (-) = 1  *“From my own experience, that doesn't resonate with me” (p12)*  (+/-) = 0  *N/A* |
| PDSS, item-21* | | **(+) = 10**  *“I don’t feel that one seems as emotive for some reason, it’s quite a direct way of getting that information and so in that way, this question is good” (p6)*  (-) = 4  *“I might want to change the phrasing to something like ‘I’ve had thoughts about hurting myself’ or something like that ‘cos ‘wanting to’ feels a bit stronger than ‘thought about it” (p1)*  (+/-) = 0  *N/A* | (+) = 6  *“It’s quite broad, but it makes absolute sense in the way it’s worded” (p2)*  **(-) = 15**  *“I think there could be some struggle to answer that question in terms of how people interpret it” (p9)*  (+/-) = 0  *N/A* | **(+) = 3**  *“Here you’ve got the chance to expand and to express yourself more and it’s a little bit less judgmental, so I would feel definitely more comfortable, even if I had to answer ‘yes” (p2)*  **(-) = 3**  *“I think if it just came out of nowhere, I think maybe a lot of people’s response is gonna be like ‘well no’ straight away, or they might get a bit upset or offended by it” (p13)*  (+/-) = 0  *N/A* | (+) = 2  *“I don’t know if it’s right to, but I would see self-harm as being part of suicide, as [suicide] being an act of self-harm” (p18)*  **(-) = 12**  *“I can't really relate to this very well, because when I think of ‘hurting myself’, I think of it as self-harm rather than killing, and I certainly didn't ‘want’ to hurt myself” (p15)*  (+/-) = 0  *N/A* |
| PDSS, item-28* | | **(+) = 12**  *“It’s just very neutral, there’s no dramatic language, I think this would have picked up on some key experiences [for me] and would have been useful information for the professionals” (p5)*  (-) = 10  *“I think that’s quite an upsetting question, it’s kind of like ‘you’re not good enough’ and I just think that would be quite triggering to see in writing there, like you’re kind of failing your baby” (p7)*  (+/-) = 0  *N/A* | (+) = 2  *“This one makes complete sense to me, and would have hit a key thought” (p5)*  **(-) = 11**  *“Thats not entirely clear, ‘cos you could feel that your baby would be better off without you, but you wouldn’t necessarily mean that’s because you wanted to complete suicide, you might just think that the baby would be better with someone else” (p11)*  (+/-) = 0  *N/A* | (+) = 4  *“I definitely think this question is an easier one to confront yourself, because it relates to wanting to protect your baby in a way… I think that there’s an element with suicidal questions- a feeling of shame of having those thoughts and so when it comes to wanting the best for your baby… even if it’s in a very negative way, it’s still something that’s easier to confront to yourself, if you know what I mean” (p6)*  **(-) = 7**  *“That kind of question would put my barriers up a little bit, it’s not something that I would find easy to admit… I think that one of peoples biggest fears when it does come to their mental health and being a new mum and stuff is the baby being taken from them, so for that question, you’re acknowledging yourself that you think the baby would be better taken- better without you, then is that gonna be a trigger for someone taking your baby [?]… yeah, I think that I’d struggle to answer that question honestly” (p8)*  (+/-) = 0  *N/A* | (+) = 0  *N/A*  **(-) = 15**  *“That one doesn't necessarily talk to me about suicide, I mean not at all actually, and I'm only just remembering that this [interview] is about suicide actually, you can say that about your baby, and you just mean ‘I can't do it, I can't do this parenting, I’m rubbish at it” (p17)*  (+/-) = 1  *“I think it would depend a bit on context, if there were a lot of questions around adoption or addiction, or quality of life, or socioeconomic situation, then I would not interpret it the same way as if there were a lot of questions around suicide and self-harm, so it would depend on the context of the questionnaire” (p18)* |
| PDSS, item-35* | | (+) = 8  *“When you’re almost playing down the language a little bit, it kind of feels less scary… [I] see it as more acceptable, like the play on the words there feels- there’s definitely something perhaps less emotive there” (p10)*  **(-) = 14**  *“I feel like that’s quite dramatic language, I’m kind of picturing someone in a film, you know, crying hysterically… maybe it’s something about ‘leaving this world’, it almost implies that there’s somewhere else to go to, and for people who have a religion, maybe that might ring true for them, but for me that doesn’t” (p3)*  (+/-) = 0  *N/A* | (+) = 2  *“To me, it is very clear that is talking about ending your life, like for me, I don’t feel that there’s ambiguity around the meaning of that and what the person’s intention would be” (p10)*  **(-) = 14**  *“This is a weird question, it conjures images of like being in a rocket ship and being shot out into space… it just feels very broad, and I don't if that's something I'd be able to answer, like if I think back to when I was in that sort of mind frame, I would be like ‘what the fuck’ [?], I would just wouldn't get it, like ‘what [?]” (p12)*  (+/-) = 0  *N/A* | (+) = 0  *N/A*  **(-) = 3**  *“The wording is still equally strong and a bit leading into one answer or the other, so I would classify that as quite strong, and you can be taken aback if you’re answer is anything else but no” (p2)*  (+/-) = 0  *N/A* | (+) = 4  *“To me, that is very clear that is talking about ending your life, like for me, I don’t feel that there’s ambiguity around the meaning of that and what the person’s intention would be, so I feel that would be like a stronger question in terms of finding someone who had seriously contemplated suicide” (p10)*  **(-) = 10**  *“It is probably just one of those phrases where you have just had enough and you just need a break, so I don’t think that would provide enough clarity, I don’t think that people would maybe acknowledge that the question that they’re asking would obviously be about killing yourself” (p8)*  (+/-) = 0  *N/A* |
| SRQ-20, item-17 | | **(+) = 23**  *“I don’t mind that question actually, because it kinda uses that phrase ‘ending your life’ which I feel more comfortable with, and I think ‘has it been on your mind’ kind of leaves it open, it’s not about ‘is it often, is it all the time’, I feel more comfortable with it because it’s slightly gentler” (p7)*  (-) = 5  *“I dunno about that one, I think maybe because it is less clinical, it does make it a little bit harder, I kinda find that a bit too personal for me to be honest, it’s about the emotion that’s behind it, so that’s a bit more challenging for me to be honest” (p8)*  (+/-) = 0  *N/A* | (+) = 4  *“It's very clear, it's clear what it means” (p17)*  **(-) = 11**  *“That feels like a really convoluted way of asking ‘have you thought of ending your own life’, I think it's about the ‘on your mind part’ it's like ‘yeah, that's on my mind as is everything else that's been on my mind’, you know, ‘the world socio political situation, climate collapse, like there's a lot on my mind’, I think it's the ‘on my mind’ part [that is confusing]” (p12)*  (+/-) = 0  *N/A* | (+) = 1  *“I’d receive this much better, if someone said to me ‘has the thought of committing suicide occurred to you often’ I’d be quite taken aback by that, like ‘ooooh, OK, err no’ but then if it was like ‘has the thought of ending your life been on your mind’, I think if it had been on my mind, I think I’d probably be more inclined to say ‘yes’, it’s more gentle and it just feels less intrusive” (p7)*  **(-) = 2**  *“Ending your life’ is a bit more emotionally provoking than the ‘suicide’, and I found anything that was emotionally provoking was quite difficult to answer, so for me, I wouldn’t want something that was gonna emotionally provoke me ‘cos then I probably might not answer it, or be unable to answer it” (p9)*  (+/-) = 0  *N/A* | **(+) = 9**  *“It could be quite helpful because it is quite soft, kind of like a gentler approach to sort of delve deeper, the language is probably a bit more accessible in this question I would say, yeah, it just feels more open” (p10)*  (-) = 0  *N/A*  (+/-) = 0  *N/A* |
| Ultra-Short, item-4 | | (+) = 8  *“That’s probably how I would want it worded to me, ‘thought’ is kinda soft enough that it’s not saying that you’re definitely gonna do it, and ‘suicide’ is a way of kinda saying what you mean without being quite as frightening as ‘death’, yeah I’m happy with that kinda wording” (p8)*  **(-) = 21**  *“I mean ‘committing suicide’ is really just not appropriate language I don't think, I know that I wouldn't say commit suicide because it's just not a crime right, so I would rather have ‘has the thought of taking your own life or ending your own life occurred to you’, so yeah, I’m not a fan” (p12)*  (+/-) = 0  *N/A* | (+) = 1  *“It’s just to the point isn't it, it's very clear what it is, yeah” (p19)*  **(-) = 20**  *“But ‘often’ what does that mean [?] ‘occurred to you often’, I think that’s the bit that’s hard, that could mean different things to different people, the fact that it’s probably occurred to you at all is probably an issue… yeah, I just don’t like that part of it, I just don’t think that’s really gonna be helpful ‘cos then if you’re someone like me and you’d thought it once you’d be like ‘no, I’ve not” (p4)*  (+/-) = 0  *N/A* | (+) = 0  *N/A*  **(-) = 11**  *“I would have concerns that they would be judging and holding and gatekeeping what needs to be defined as ‘often’, and I never say ‘commit suicide’, I think that could even subconsciously affect a woman, like we hear that phrase and even if you’re not consciously aware of the history of the language, I still think it has that affect because it’s so embedded in our culture, somehow we understand it, so yeah, the other ones are definitely easier to answer than anyone asking me ‘are you thinking of committing suicide’, it’s not a crime” (p5)*  (+/-) = 0  *N/A* | **(+) = 6**  *“The word ‘committing’ is giving you more of the action, so it’s getting a little bit closer to the actual thinking of it in terms of doing it, maybe going to places where you think you might commit the suicide, buying anything that you might want to assist with it, so there’s the thought of the actual doing it, but then there’s the thought of the process of how you would need to do it as well, and it’s all relevant” (p9)*  (-) = 0  *N/A*  (+/-) = 0  *N/A* |

*Notes:* (i) symbols (+, -, +/-) represent (+) positive, (-) negative, (+/-) or neutral/indifferent coding instances.; (ii) numerical values represent the number of unique (+), (-), or (+/-) coding instances per item/construct, and not the number of participants; (iii) codable data was not available from all participants for all items/constructs, and some participants may have provided more than one unique comment per item/construct (e.g., one participant may have made two (+) comments about different aspects of one item, or one (+) and one (-) comment); (iv) **bold** indicates the highest number of coding instances for the item/construct; (v) *N/A* = no codable data/quotations were identified for this item/construct; (vi) (p1), (p2), etc, represent the anonymous participant identifiers for each quote. (vii) *** measure has been validated in perinatal populations.

*Measures:* BDI = Beck Depression Inventory (68); EPDS = Edinburgh Postnatal Depression Scale (19); IDAS = Inventory of Depression and Anxiety Symptoms (65); PDSS = Postpartum Depression Screening Scale (66); PHQ-9 = Patient Health Questionnaire-9 (20); SRQ-20 = Self-Reporting Questionnaire-20 (67); Ultra-Short = Ultra-Short Maternal Mental Health Screen (69).
