# Supplementary material for: Acceptability and content validity of suicidality screening items: a qualitative study with perinatal women
Source: Front Psychiatry. 2024 Apr 11;15:1359076. doi: 10.3389/fpsyt.2024.1359076 (PMC11044181; doi:10.3389/fpsyt.2024.1359076)
Supplement: Supplementary file 2 [file Table_2.docx]

**Supplementary Table 2**

Number of positive, negative, and neutral/indifferent coding instances for the suicide-related item response options and recall periods, with illustrative participant quotes

TFA construct

| Measure | Response options | Recall period |  |
| --- | --- | --- | --- |
| BDI, item-9 | | **(+) = 11**, (-) = 10, (+/-) = 0  (+) *“The scale is quite interesting actually, quite helpful, because it gives people a chance to really reflect on the thoughts they've been having and think about like the nature of them, like ‘is it this, is it- how do I actually feel about this’, ‘cos I guess in a way, actually it's quite helpful for people to have a moment to think, you know, ‘how serious is this’ and reflect on it, which I think is quite good, and that’s quite helpful because it gives much more information” (p21)*  (-) *“It feels a bit weird not having a question there, so it feels a bit uncomfortable, it feels a bit like a quiz, it’s really weird” (p7)*  (+/-) *N/A* | (+) = 1, **(-) = 8**, (+/-) = 0  (+) *“The past seven-days is OK” (p1)*  (-) *“Again, you probably don’t need the past seven-days, you could do it ‘since pregnancy’ if you were trying to identify someone” (p6)*  (+/-) *N/A* |
| EPDS, item-10 | | **(+) = 14**, (-) = 8, (+/-) = 3  (+) *“It would be clear to me what the answers are, you could probably easily pick one of them” (p18)*  (-) *“I think when you’re in that mindset it might be really difficult to kind of decide, where you feel you’d fit into that, which could lead to that person thinking… ‘oh, I don’t wanna say’ because it’s hard trying to fit yourself into a box” (p9)*  (+/-) *“I don’t know that if somebody’s feeling like they wanted to hurt themselves [they] would necessarily think about the kind of frequency of that thought, I don't know” (p17)* | (+) = 11, **(-) = 19**, (+/-) = 2  (+) *“I think seven-days is probably about right, when you’re in that first month or two, seven-days is a really long time period, you’re awake for almost 24-hours of each of those seven-days and so having a fairly short timescale still captures quite a lot of variability I think, so yeah, seven-days for perinatal feels about right to me” (p3)*  (-) *“I actually think it should be over a longer period because in a way it’s quite dismissive isn’t it… I’d feel a bit like ‘maybe I shouldn’t say anything’ or ‘maybe it doesn’t matter if it was over seven-days ago ‘cos it’s not as important” (p7)*  (+/-) *“I mean maybe what might be helpful is ‘seven-days’ and then in the ‘past six-months or a year” (p18)* |
| IDAS, item-7^1^  IDAS, item-9^1^  IDAS, item-14^1^  IDAS, item-15^1^  IDAS, item-41^1^  IDAS, item-43^1^ | | (+) = 4, **(-) = 27**, (+/-) = 0  (+) = 4, **(-) = 27**, (+/-) = 0  (+) = 6, **(-) = 23**, (+/-) = 0  (+) = 3, **(-) = 25**, (+/-) = 0  (+) = 3, **(-) = 25**, (+/-) = 0  (+) = 2, **(-) = 26**, (+/-) = 0  (+) *“I quite like the ‘extremely’ bit, ‘cos you get a sense of how it’s impacting someone's life, and you know, ‘OK, yeah, a little bit’ rather than the ‘several days’, so I think I prefer the scale on this one” (p20)*  (-) *“I could see me getting a bit confused here, like have you asked me to quantify them, like ‘thoughts of, like how may’, but then ‘extremely’ makes me think of intensity, so those answers confuse me because I think ‘well, what do you want’, are you asking about how many thoughts, how often, or are you asking like how intense they are [?], those are two very different things, and this feels a bit jumbled between intensity and frequency, and I’m not quite sure how I would answer that” (p5)*  (+/-) *N/A* | (+) = 3, **(-) = 9**, (+/-) = 0  (+) = 3, **(-) = 9**, (+/-) = 0  (+) = 3, **(-) = 9**, (+/-) = 0  (+) = 3, **(-) = 9**, (+/-) = 0  (+) = 3, **(-) = 9**, (+/-) = 0  (+) = 3, **(-) = 9**, (+/-) = 0  (+) *“I mean it’s still an OK timescale, you would definitely be able to use that timescale, you could fathom the answer, and it will capture more people than just the last seven-days” (p18)*  (-) *“Again, I think putting kind of weeks on it is quite arbitrary, I think it should just be kinda a generalised period of ‘pregnancy, post-birth’ or that kind of thing” (p8)*  (+/-) *N/A* |
| PHQ-9, item-9 | | **(+) = 13**, (-) = 10, (+/-) = 0  (+) *“The frequency scale is more meaningful I think, yeah, I would understand what I was being asked there and be able to answer it accurately ‘cos this one gives you an option of ‘not at all’ which is good, and then obviously going up to ‘nearly every day” (p11)*  (-) *“I hate these answers, I hate ‘more than half the days’ I mean what are you supposed to do, calculate them [?], it doesn't make sense to me, I understand ‘not at all’ and ‘nearly every day’ I get those two, but ‘several days’, and ‘more than half the days’ I've never really understood that and I don't like the way it's phrased, it’s too much of a thinker, you're not going to know exactly which days you have and haven't, so it's either ‘sometimes’ or ‘not at all’ or ‘every day’, isn't it [?]” (p15)*  (+/-) *N/A* | (+) = 3, **(-) = 11**, (+/-) = 0  (+) *“That [timescale] would have worked better for me” (p5)*  (-) *“I would like expand that timeframe as I think you could miss out on some people by limiting yourself to two-weeks” (p13)*  (+/-) *N/A* |
| PDSS, item-7^2^  PDSS, item-14^2^  PDSS, item-21^2^  PDSS, item-28^2^  PDSS, item-35^2^ | | (+) = 6, **(-) = 26**, (+/-) = 0  (+) = 8, **(-) = 22**, (+/-) = 0  (+) = 10, **(-) = 22**, (+/-) = 0  (+) = 9, **(-) = 23**, (+/-) = 0  (+) = 9, **(-) = 21**, (+/-) = 0  (+) *“I like that you get to ‘feel’ the answers, so ‘strongly agree’, it's like you're strongly feeling that, or you're ‘strongly agreeing’ with that” (p15)*  (-) *“That scale’s just really strange isn’t it, it’s like quite cold, it just feels very clinical, like would you really tick ‘oh, strongly agree or agree’ if you were in that headspace, I mean I’m not in that headspace and I would feel a bit uncomfortable about answering like ‘disagree or strongly disagree’, and ‘neither disagree or agree’ just feels very weird as well, I don’t know if I’d want to see this scale” (p7)*  (+/-) *N/A* | (+) = 6, **(-) = 16**, (+/-) = 5  (+) = 5, **(-) = 16**, (+/-) = 3  (+) = 5, **(-) = 16**, (+/-) = 3  (+) = 6, **(-) = 15**, (+/-) = 3  (+) = 5, **(-) = 15,** (+/-) = 3  (+) *“The past two-weeks again is a fairly short timeframe, so you’ve got still quite clear memories of what’s been happening” (p2)*  (-) *“I do feel like the timeframe still feels quite short, I don't think memory is an issue really, because people do generally recall if they have quite serious suicidal thoughts, so I just think the timeframe is a bit of a funny one and I almost wonder if it needs to be like, you know, ‘since you've been pregnant or since…’ ‘cos it's more about the pregnancy itself than it is about the time, so I’m wondering that for all the questions really” (p21)*  (+/-) *N/A* |
| SRQ-20, item-17 | | **(+) = 9**, (-) = 3, (+/-) = 1  (+) *“I think that almost ‘yes/no' questions are a bit easier to verbalise, I mean the frequency is useful diagnostically, but beyond therapy, I’d have an initial one which is just ‘have you had these thoughts” (p6)*  (-)*“It’s almost, if you’re answering ‘yes’, then it feels like you’re constantly thinking about it, I don’t think that gives you enough kinda options, its either ‘yes, it’s on your mind’, like that’s it, or it’s not, like a fleeting thought that you’re having every now and again, so I think that the ‘yes or no’ is probably a bit divisive for that one” (p8)*  (+/-) *“I might change the answer options to some sort of frequency-based scale ‘cos you’re not getting enough information, although it’s definitely not as much of an issue as it was with the previous question because answering ‘yes’ kind of implies that its once or at least once, which is already enough to continue on with more detail” (p13)* | (+) = 2, **(-) = 10**, (+/-) = 0  (+) *“That’s sort of got like a slightly longer- like the ‘past month’, I think that feels a bit more [appropriate] ‘cos it’s over a longer period of time, so yeah” (p7)*  (-) *“Over the past month’ I mean that’s a doable timeframe, but it will depend on what people are trying to get out of it, and I don’t like there being cut offs, so I would prefer if it was ‘ever” (p18)*  (+/-) *N/A* |
| Ultra-Short, item-4 | | **(+) = 11**, (-) = 8, (+/-) = 0  (+) *“I think ‘yes or no’ is probably better than the previous options, just because if there is a thought there, then it obviously needs to be looked at, regardless of the frequency or the severity of it” (p9)*  (-) *“Suicide’ is a strong word, so when you put it this way and it’s just a ‘yes or no’ maybe it is a bit intimidating so having ‘never, at least once, or often’ could allow women to open up and say ‘yeah, maybe at least once’ so it allows women to be a little bit less protective of themselves in opening up without feeling too judged I guess, that’s how I would feel” (p2)*  (+/-) *N/A* | **(+) = 8**, (-) = 7, (+/-) = 1  (+) *“I think the timescale is probably more helpful than in previous questions because it’s obviously longer and that probably takes into account some fluctuations of general life and mood and mental health and things like that” (p10)*  (-) *“I would even consider having it for a longer period to be honest, because I would like to answer through the whole pregnancy, from the day I found out until now, because these type of questions are something that I’m not gonna forget” (p2)*  (+/-) *“A month is maybe a little bit better, I feel like it’s not quite as restrictive, I wouldn’t have quite as much of an issue as saying it was in the last seven-days or if it was in the last fortnight, but again, I still think that ‘recently’ or something like that would be a bit better” (p8)* |

*Notes:* (i) superscript numbers (^1, 2^) indicate the same response options and recall periods (e.g., subscale); (ii) symbols (+), (-), (+/-) represent (+) positive, (-) negative, (+/-) or neutral/indifferent coding instances.; (iii) numerical values represent the number of (+), (-), or (+/-) coding instances for the item response options and recall period, and not the number of participants; (iv) codable data was not available from all participants for all item response options or recall periods, and some participants may have provided more than one unique comment per item response option or recall period (e.g., one participant may have made two (+) comments about different aspects of a response option, or one (+) and one (-) comment); (v) some participants made one or more comments that applied to all PDSS and/or IDAS item response options and/or recall periods; (vi) only one (+), (-) and (+/-) illustrative quote has been provided for the PDSS and IDAS subscale items and recall periods; (vii) **bold** indicates the highest number of coding instances for the item response option and recall period; (viii) *N/A* = no codable data/quotations were identified for this response option or recall period; (ix) (p1), (p2), etc, represent the anonymous participant identifiers for each quote.

*Measures:* BDI = Beck Depression Inventory (68); EPDS = Edinburgh Postnatal Depression Scale (19); IDAS = Inventory of Depression and Anxiety Symptoms (65); PDSS = Postpartum Depression Screening Scale (66); PHQ-9 = Patient Health Questionnaire-9 (20); SRQ-20 = Self-Reporting Questionnaire-20 (67); Ultra-Short = Ultra-Short Maternal Mental Health Screen (69).
